# Y chromosome instability in the early mouse embryo

**DOI:** 10.1126/sciadv.aej3098

**Published:** 2026-11-06

**Authors:** Agata P. Zielinska, Richelle Rietdijk, Valdone Maciulyte, Aurelien Courtois, James M. A. Turner

**Affiliations:** Sex Chromosome Biology Laboratory, Francis Crick Institute, 1 Midland Road, London NW1 1AT, UK.

## Abstract

The Y chromosome lacks canonical centromeric repeats necessary for high-fidelity chromosome segregation. In adult tissues, this causes Y loss and predisposes to disease. However, its impact on embryogenesis is unknown. Here, we identify the first mouse embryonic mitoses as a window during which the Y centromere is unusually weak. This vulnerability impairs kinetochore strength and the Y becomes preferentially trapped at the spindle pole, resulting in missegregation. To study the impact of Y loss, we generate an aggregation model to track specific aneuploidies in mosaic embryos. Loss of the Y slows down cell division and compromises embryo size. Our findings reveal a developmental window during which the Y is especially vulnerable to loss, with implications for male sex determination and the origin of Turner syndrome (XO).

## INTRODUCTION

Y chromosome loss is the most frequent chromosomal abnormality acquired by adult men and is associated with an increased risk of various diseases of old age, including cancer, cardiac fibrosis, and dementia (*1*, *2*). Although Y-null cells may accumulate in adult tissues because of the relative functional tolerance of Y loss, evidence indicates that intrinsic properties of the Y centromere make it especially prone to missegregation during mitosis (*3*–*6*). Notably, in both mice and humans, the Y chromosome uniquely lacks the CENP-B motif at its centromere (*7*, *8*), instead relying exclusively on the histone H3 variant CENP-A for kinetochore assembly (*9*–*12*). Given that the kinetochore orchestrates the interactions with spindle microtubules as the cell divides (*13*–*15*), the reliance on a singular pathway for its assembly elevates the Y chromosome’s susceptibility to missegregation in somatic cells and tissue culture models (*3*, *6*, *16*, *17*).

While Y chromosome loss in adults is disadvantageous, its missegregation during early embryogenesis would have far more profound consequences, particularly for male sex determination and the origins of Turner syndrome (*18*–*21*). Since the Y chromosome specifies male sex, proper segregation must be maintained at least until approximately 10 days postconception in mice and 6 weeks in humans, when sex chromosome complement is translated into phenotypic sex (*20*). While Turner syndrome affects approximately 1 in 1000 to 2500 births, an estimated 1 to 5% of all conceived embryos lack a sex chromosome (*1*). Intriguingly, whereas most embryonic aneuploidies involve maternal chromosomes and increase with advancing female age (*22*–*24*), ∼74% of sex-chromosome monosomies arise from loss of the paternal chromosome (*25*, *26*). Given the high early lethality of embryos lacking a sex chromosome (*1*), the true frequency with which Turner syndrome embryos begin development as XY remains uncertain. This raises some critical questions: How does the inherently vulnerable Y chromosome navigate this precarious developmental window, and could the early loss of a sex-chromosome mean that a larger fraction of Turner syndrome conceptions originate from XY embryos than currently appreciated?

The first mitotic division of the embryo may present a particular challenge for centromere architecture. In mice, egg and sperm chromatin carries different amounts of CENP-A (*27*, *28*). After fertilization, CENP-A levels must then equalize across parental chromosomes (*29*, *30*). This requirement challenges the typical mechanism of centromere maintenance, which operates by keeping total CENP-A levels constant across cell cycles through a 1:1 stoichiometric deposition driven by preexisting CENP-A (*11*, *31*). As a fraction of CENP-A is lost during mouse spermatogenesis, the paternal pronucleus in the zygote starts with lower CENP-A levels in comparison to the maternal pronucleus (*30*). The restoration of CENP-A levels in the zygote appears to depend primarily on a CENP-B–dependent pathway and not on the number of preexisting CENP-A molecules (*28*). The ability of the Y centromere, which lacks CENP-B, to recover after fertilization remains unclear, particularly since 75% of Y chromosomes in human tissue culture cells fail to accumulate detectable CENP-A when starting with no chromatin-bound CENP-A (*4*). How the Y chromosome overcomes these challenges in early embryogenesis—rebuilding a functional centromere within a single cell cycle postfertilization, while starting with diminished CENP-A bound to the paternal chromatin (*30*)—remains a fundamental and unresolved question.

One possibility is that the distinctive physiological environment of early embryonic cells fosters efficient Y chromosome centromere assembly, providing a critical developmental safeguard against sex chromosome aneuploidy. Alternatively, Y chromosome missegregation may occur more frequently than previously recognized but may be buffered by counterselection. In this study, we combine advanced microscopy techniques, embryo assays, and genetic models of defined sex chromosome aneuploidy to study sex chromosome behavior in early mouse embryos, and investigate how early development copes with errors that do occur.

## RESULTS

### The two X chromosomes in the female zygote incorporate different amounts of CENP-A despite shared centromeric DNA sequence

To study how chromatin epigenetic memory affects centromeres with distinct properties, we developed a strategy to simultaneously analyze CENP-A levels, parent of origin (maternal versus paternal; Fig. 1A), and sex chromosome complement (XX versus XY) at the first embryonic metaphase (fig. S1A). We used a two-step approach, where we first imaged CENP-A and trimethylated histone H3 lysine 9 (H3K9me3) that preferentially labels maternal chromosomes (Fig. 1B) (*32*, *33*), followed by DNA fluorescence in situ hybridization (FISH) to ascertain sex chromosome identity. To ensure that we captured all embryos at metaphase, we required a technique to synchronize them, given that embryos enter the first mitosis asynchronously (∼5-hour window) and the metaphase stage is brief (∼65 min) even in zygotes. To achieve this, we used APCin, a small inhibitor of the anaphase promoting complex (*34*, *35*). We confirmed with light-sheet live microscopy that APCin effectively prevented anaphase (efficiency 100%; fig. S1, B to D). Our assay allowed reliable analysis of 94.8% of centromeres per cell (fig. S1E), offering a technical advantage over previous methods for embryos and enabling precise study of the centromeric architecture of individual sex chromosomes.

**Fig. 1.**
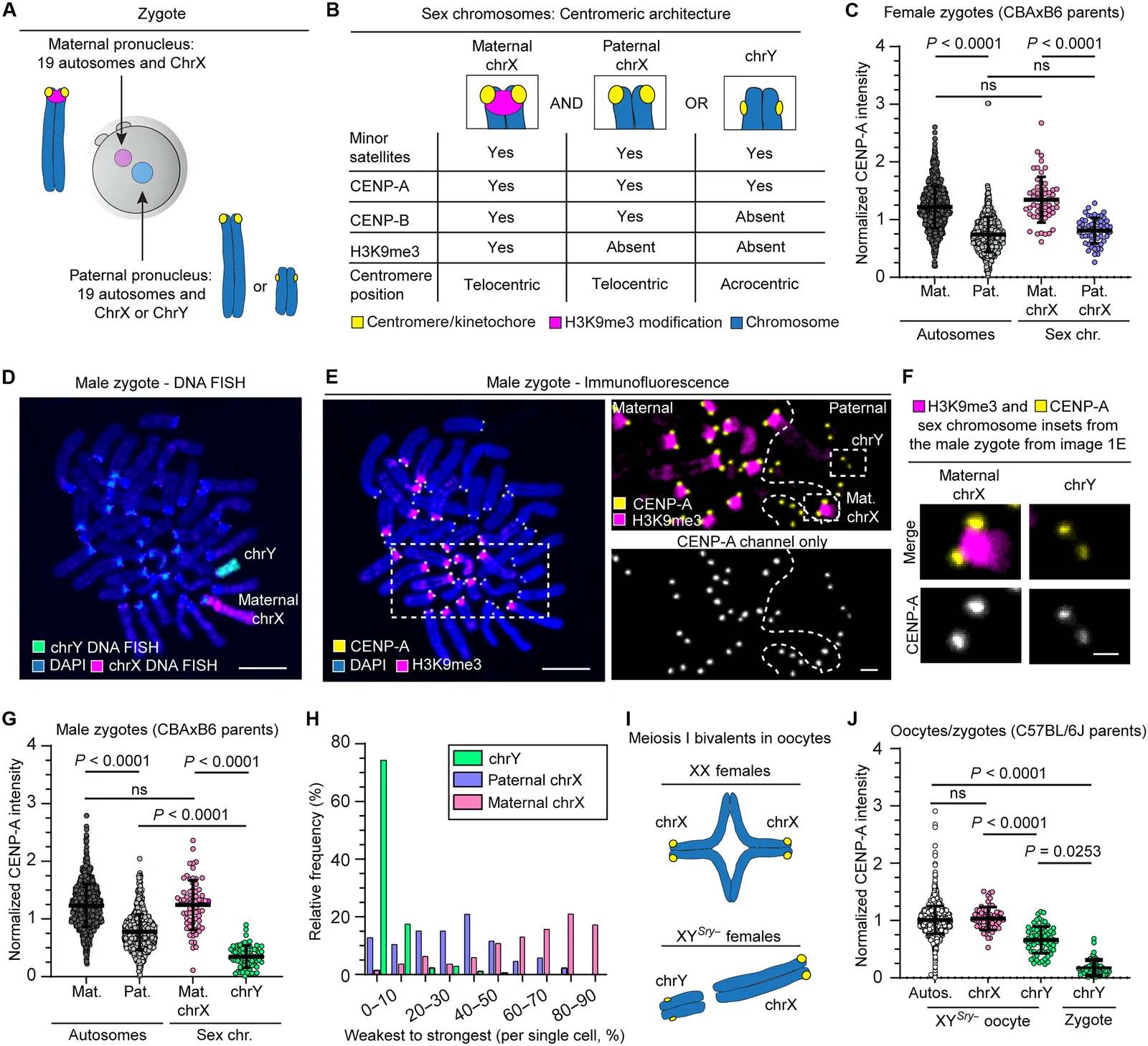
In zygotes, the Y chromosome is among the 10% weakest centromeres and parent of origin determines the strength of the X centromere. (**A**) Schematic of parental chromatin separation in a mouse zygote. (**B**) Sex chromosome centromere architecture. (**C**) Quantification of normalized CENP-A fluorescence intensity of individual centromeres in female zygotes (*Ν* = 32 from 3 mice) from CBAxC57BL/6J F1 parents. Centromeres analyzed: *n* = 1221 maternal autosomes; 1190 paternal autosomes; 56 chrX maternal; 57 chrX paternal. (**D**) DNA FISH labeling of sex chromosomes from (E). (**E**) Maximum intensity projection of chromosomes in a male mouse zygote from CBAxB6 F1 parents. H3K9me3^+^: maternal, H3K9me3^−^: paternal. (**F**) Insets: sex chromosome centromeres from (E). (**G**) Quantification of normalized CENP-A fluorescence intensity of individual centromeres in male zygotes (*N* = 35 from 3 mice) from CBAxB6 F1 parents. Centromeres analyzed: 1308 maternal autosomal, 1267 paternal autosomal, 66 chrX, 68 chrY. (**H**) Centromeres classified by CENP-A intensity into 10 equal bins, from the weakest 10% to the brightest 10% in each zygote. *N* = 25 mice; *n* = 180 chrY, 74 chrX paternal, and 225 chrX maternal. (**I**) Schematic of sex chromosomes in meiosis I oocytes from wild-type XX females (top) and XY *^Sry-^* females (bottom). (**J**) Quantification of normalized CENP-A fluorescence intensity in XY *^Sry-^*oocytes, as compared to the chrY in zygotes of the same strain (C57BL/6J, replotted from fig. S2E). Analyzed: 69 oocytes from 6 mice (2316 autosomal, 61 chrX, and 69 chrY centromeres) and 27 zygotes from 6 experiments (52 chrY centromeres). Boxes mark regions that are magnified in insets. In graphs, each dot represents a single centromere. Scale bars, large images 10 μm (D and E), insets 2 μm, (E) or 1 μm (F). Statistical significance was evaluated using Kruskal-Wallis test (C, G, and J). Error bars show means ± SD.

We first focused on sex chromosomes in female zygotes (Fig. 1C and fig. S1, F to I), given that X and Y share the same evolutionary ancestry (*21*). In particular, we examined whether the two X chromosomes within the same cell carry different amounts of CENP-A despite having the same theoretical capacity to incorporate it. Given that the two homologous chromosomes share DNA centromeric sequences and hence recruit comparable amounts of CENP-B, the differences in their strength will depend on the amount of CENP-A incorporated (*8*, *11*). Centromeres of paternal autosomes in female embryos from CBAxB6 F1 hybrid parents had on average fewer CENP-A than maternal autosomes (paternal to maternal ratio = 0.61; Fig. 1C and fig. S1F). This is in line with previous work in intact nuclei (*30*) and mice heterozygous for CENP-A (*28*). While the X chromosome did not differ from the average autosome of the same parental origin (Fig. 1C), there was a significant difference in the strength of the two X centromeres, with the paternal X chromosome incorporating 39.9% fewer CENP-A than the maternal X chromosome (Fig. 1C and fig. S1, G and H). The paternal X chromosome was also significantly weaker than the maternal X in the inbred C57BL/6J mice (fig. S1I and movie S1). This suggests that during the first mitotic division, epigenetic footprints linked to parental origin can greatly influence the strength of the two homologous X chromosomes in the same cell, regardless of their centromeres sharing the same genetic characteristics. As a result, the one-cell stage female embryo must be equipped to reliably segregate both the weak paternal X centromere and the 1.66 times stronger maternal X (Fig. 1C and movie S1).

### In zygotes, the Y chromosome incorporates the least CENP-A

Next, we examined CENP-A levels in male zygotes (Fig. 1, D to H). Given that the Y centromere is fully reliant on CENP-A (Fig. 1B), we expected that CENP-A levels on the Y centromere would approximate other paternal chromosomes. Unexpectedly however, our analysis of male embryos showed that the Y centromere had profoundly lower CENP-A levels even when compared to the weaker paternal autosomes only (34.6 versus 77.7% fluorescent signal intensity as compared to an average chromosome, Fig. 1, D to G, and movie S2). This trend was consistent when analyzed on an embryo-by-embryo basis: In most embryos (75.0%), the Y centromere was among the 10% weakest staining for CENP-A (Fig. 1H; comparison across the 40 chromosomes per zygote). This effect was not strain specific. In zygotes from the inbred C57BL/6J strain (fig. S2, A to F), the Y centromere was even weaker than in those from the CBAxB6 F1 hybrid parents (Fig. 1G), with CENP-A levels at only 17.5% of that seen in other chromosomes (fig. S2E). We confirmed this result using an alternative CENP-A antibody, finding that CENP-A levels on the Y chromosome were 11.8% of that on other chromosomes (fig. S2, C, D, and F). These results are in contrast to human tissue culture cells, where the Y chromosome has been studied in detail and was found to recruit only slightly fewer (*36*, *37*) CENP-A than autosomes (∼82% of autosome level), with some reports indicating no significant difference (*3*). The reduced levels of CENP-A in zygotes were unlikely to be a labeling artefact, as the opposite pattern was observed for other proteins. For instance, we showed that the centromeric cohesin protector, Shugoshin2 (SGO2) (*38*), was more abundant at the zygotic Y chromosome than at the X or autosomes (fig. S2, G to I). Together, these data suggest that the only chromosome that relies entirely on CENP-A for its kinetochore is inefficient at building up CENP-A levels for the first mitotic division.

In addition to adequate CENP-A incorporation, chromosomes rely on an even CENP-A distribution between the two sister chromatids to form microtubule bundles of equal sizes and align stably at the spindle equator. Uneven distribution of CENP-A between sister chromatids during anaphase could lead to unequal microtubule pulling (fig. S3A), increasing the risk of chromosome nondisjunction and aneuploidy. We found that 32.4% of Y chromosomes (versus <6.5% of all autosomes or X chromosomes) exhibited a strong asymmetry between sister centromeres (fig. S3, B and C). These data were in contrast to human somatic cells, where CENP-A distribution across the two sisters of autosomes and the Y chromosome is nearly equal (*39*). Therefore, CENP-A incorporation is both reduced and asymmetric on the mouse Y centromere, which can create a dual challenge to accurate chromosome segregation.

### Y transmission through the female germline improves CENP-A incorporation

We then investigated whether the unique packaging of the Y chromosome in the sperm head could contribute to its centromere being particularly weak. To test this idea, we developed a strategy to transmit the Y chromosome through the female germline instead, circumventing the chromatin changes that occur during male gametogenesis. We used XY*^Sry−^* mice (*40*) (Fig. 1I), which develop ovaries since their Y chromosome lacks the *Sry* gene that determines male biological sex (*19*, *21*). Within the XY*^Sry−^* oocyte (fig. S3D), the Y centromere also incorporated fewer CENP-A than autosomes; however, the levels were increased to 66.1 versus 17.5%, compared to the Y chromosome in zygotes of the same strain (Fig. 1J). As a result, when assembled in the oocyte, the Y centromere is 3.8 times stronger than in the zygote. This effect was not an artifact of the *Sry* deletion because male zygotes derived from XY*^Sry-^ Sry* transgenic fathers (*41*) incorporated similar levels of CENP-A (fig. S3, E to H) as male zygotes from XY fathers (fig. S2E) of the same strain. These results suggest that transmission through the sperm creates a vulnerability for CENP-A incorporation to the Y chromosome.

### The Y centromere is inherently weak in embryos from both young and older mothers

When the Y centromere is assembled in the zygote, it relies on preexisting maternal CENP-A protein pool derived from the oocyte (*42*). This is because zygotes are transcriptionally silent (*30*, *43*), their cytosolic pool of *CENP-A* mRNA is small (*44*), and blocking translation does not lower CENP-A levels in the paternal pronucleus (*30*). Since females are born with all their oocytes, female aging corresponds to oocyte aging. Despite the remarkable stability of the chromatin-associated CENP-A nucleosomes (*42*, *44*–*46*), their levels decline up to ∼70% by the end of the reproductive life span (*47*). We hypothesized that the low starting levels of CENP-A in aged oocytes could further impair the ability of the Y chromosome to incorporate CENP-A after fertilization (fig. S4A).

To investigate this, we compared zygotes from young (6 to 9 weeks) and reproductively older females (52 to 65 weeks). We confirmed that this corresponds to reproductively aged females, as shown by a reduced ovarian pool (39 versus 9 oocytes per animal) and reduced centromeric CENP-A levels in metaphase II oocytes (28.5% of the levels seen in young oocytes; fig. S4, B and C). Thus, in agreement with previous studies, aging leads to a loss of chromatin-associated CENP-A in oocytes (*44*, *47*).

Unexpectedly, we found no difference in absolute centromeric CENP-A intensity per embryo between young and aged zygotes (fig. S4, D and E), and the maternal chromatin maintained its stronger centromere status (fig. S4, F and G) despite originating from weak and aged metaphase II centromeres (fig. S4, B and C). In line with these findings, maternal age had no influence on the Y centromere strength (fig. S4H). Collectively, our data show that female centromeres weakened by oocyte aging recover before the first mitosis. Thus, the negative impact of aging on maternal centromeres is confined to oocyte meiosis as neither maternal centromeres nor the Y chromosome in zygotes are adversely affected by the reduced CENP-A pool brought about by female aging.

### The zygotic weakness of the Y centromere recovers after the embryonic genome is reactivated

We then investigated whether the pronounced Y centromere weakness we found is confined to a specific developmental window. We predicted that recovery could occur after embryonic genome activation (EGA) as parental asymmetries at the whole cell level equalize by the four-cell stage (*28*). In mice, major EGA occurs after the two-cell stage, so we compared embryos pre-EGA (zygote and two-cell) and post-EGA (four-cell stage or eight-cell stage; Fig. 2, A and B). The CENP-A ratio (Y/X CENP-A fluorescent intensity) remained the same between the zygotes and two-cell stage embryos, but it increased by the four-cell stage (Fig. 2B).

**Fig. 2.**
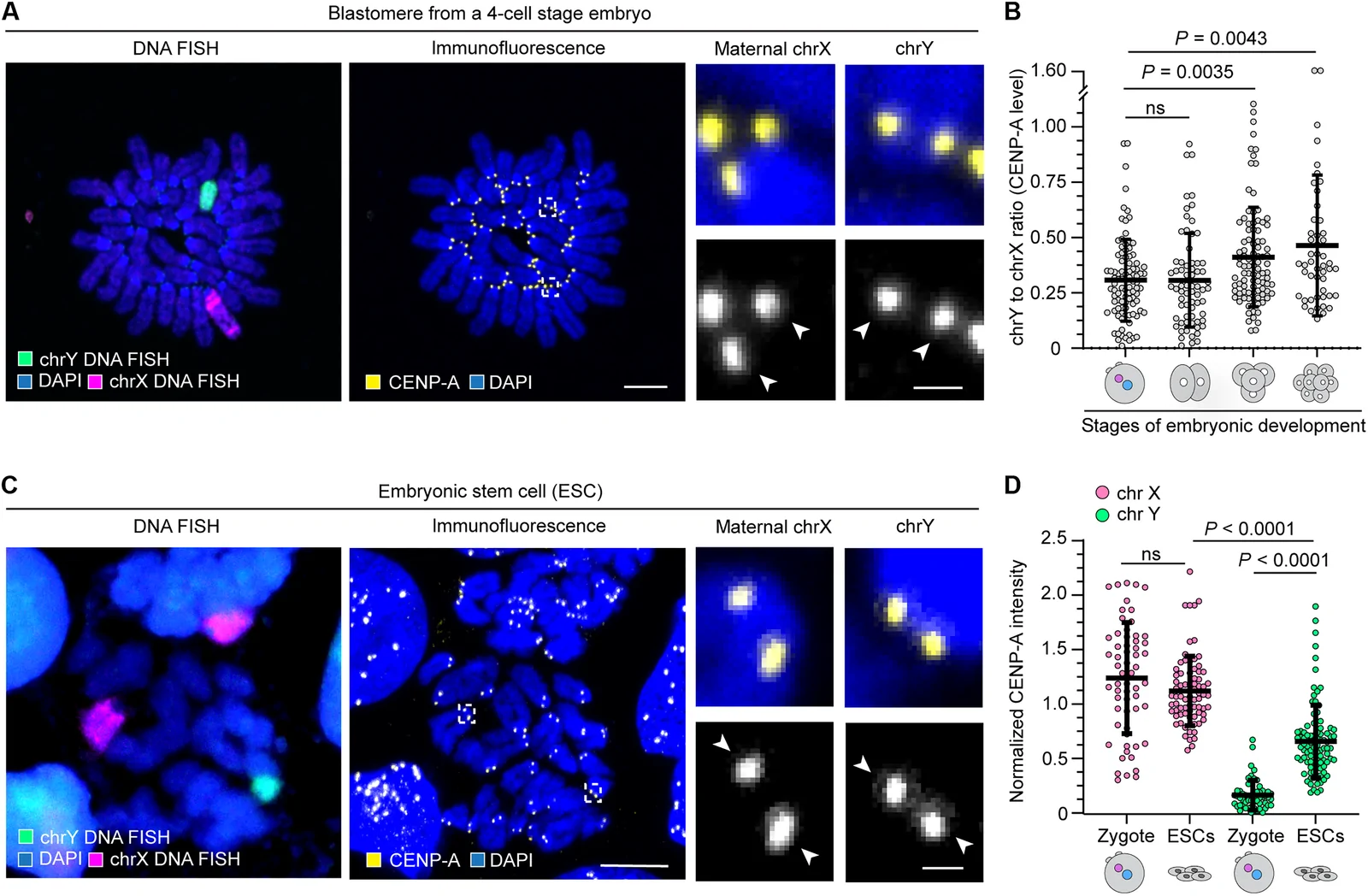
The Y centromere strength recovers as embryonic development advances. (**A**) Maximum intensity projection of a chromosome spread from a four-cell embryo blastomere (CBAxC57BL/6J F1 hybrid parents). (**B**) Quantification of the chrY to chrX normalized CENP-A fluorescence intensity ratio at various developmental stages (zygote, two-cell, four-cell, and eight-cell) in embryos from CBAxC57BL/6J F1 hybrid parents. Data for zygotes ares replotted from Fig. 1G. The number of centromere pairs analyzed is *n* = 95 for one-cell, 68 for two-cell, 95 for four-cell, and 49 for eight-cell embryos. (**C**) Representative maximum intensity projection of a chromosome spread of a mouse ESC from the C57BL/6J strain, showing DNA FISH and immunofluorescence. (**D**) Quantification of normalized CENP-A fluorescence intensity for chrX (magenta) and chrY (green) in ESCs compared to zygotes (all from C57BL/6J mice). Each dot represents an individual centromere. The analysis included *n* = 170 centromeres from ESCs (three experimental repetitions) and 57 centromeres from 27 zygotes (the latter data replotted from fig. S2E). Boxes mark regions that are magnified in insets. Arrowheads highlight individual centromeres of sex chromosomes. Scale bars, overviews (10 μm); insets (2 μm). Statistical significance was evaluated using Kruskal-Wallis test (B and D). Error bars show means ± SD.

To determine whether CENP-A levels in post-EGA embryos match the Y centromere strength in typical mitotically dividing cells, we looked at mouse embryonic stem cells (ESCs) from the same mouse strain (Fig. 2C). While the Y chromosome was also among the weakest centromeres in ESCs, it was 3.8 times stronger than in the zygote (66.8 versus 17.5%, all C57BL/6J mouse strain; Fig. 2D, green) and almost equal to that at the four-cell stage (Fig. 2B). We found no significant difference in the strength of the X centromere under these conditions (Fig. 2D, magenta). Together, the increase in Y’s strength with development may explain why the Y chromosome missegregates 24 times more frequently in zygotes than in eight-cell stage embryos in BALB/cWt mice (*48*), which experience high level of sex chromosome aneuploidy due to a mutation that further reduced the Y centromere size (*49*). Hence, there is a paradox—our findings show that the Y chromosome is weakest at a developmental stage when the consequences of a single missegregation event are most severe as Y loss during the first embryonic division can lead to sex reversal (*19*).

### The weak Y centromere builds a small kinetochore that is under less tension from spindle microtubules

We then focused on whether the weak Y centromere predisposes zygotes to chromosome missegregation. Given that the zygotic Y centromere lacks the CENP-B box and we found has severely diminished CENP-A levels, we predicted that kinetochore formation may be compromised. We examined the outer kinetochore component Hec1 (Fig. 3A), which interacts directly with spindle microtubules (*31*). Consistent with our prediction that the Y kinetochore would be weaker, Hec1 fluorescence intensity on the Y chromosome in zygotes from CBAxB6 F1 hybrid parents was only 41.8% of that in autosomes (Fig. 3B). This trend was consistent in inbred C57BL/6J mice (fig. S5, A and B). Thus, the weak Y centromere does translate into a weak Y kinetochore in zygotes.

**Fig. 3.**
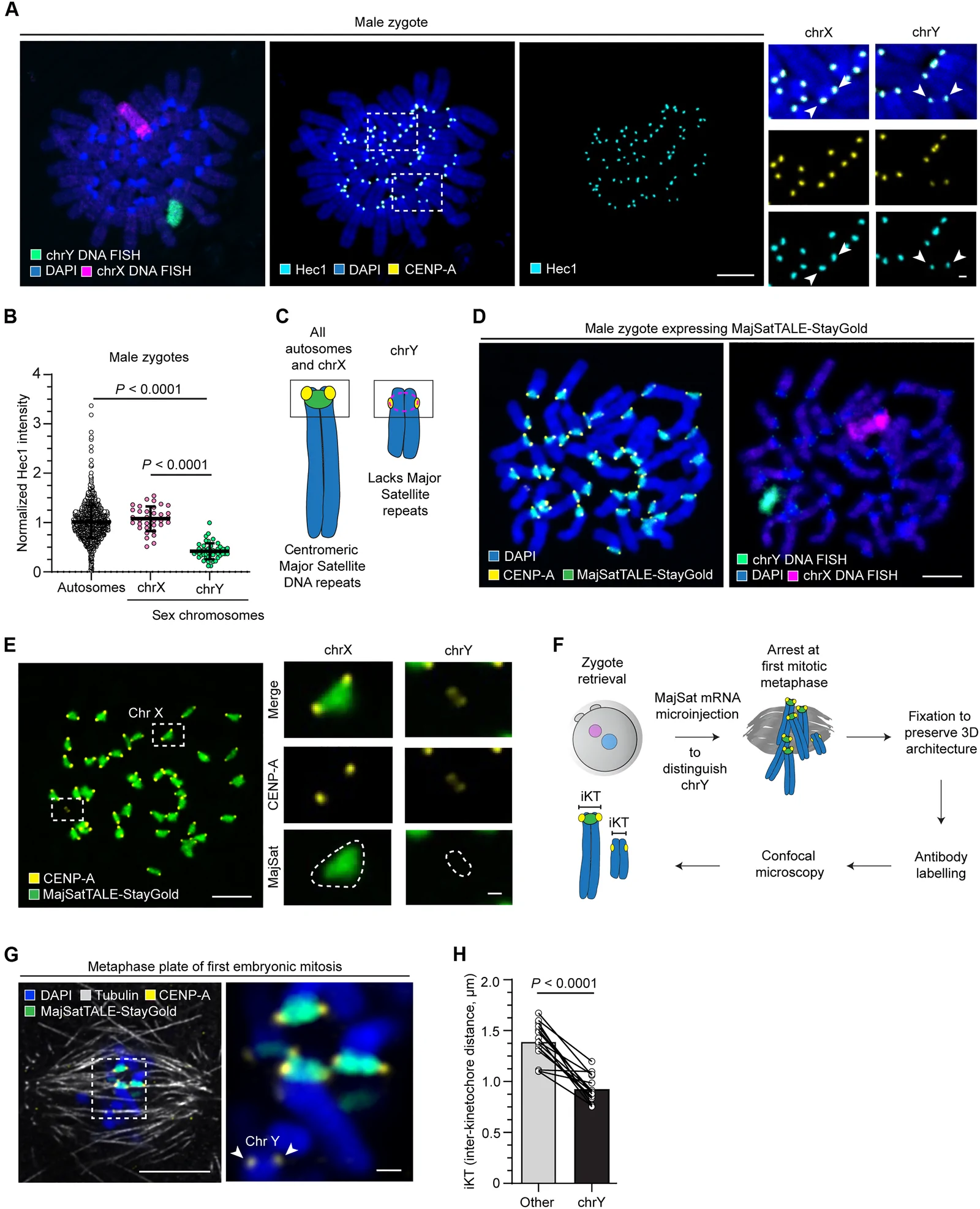
The weak centromere of the zygotic Y translates to a weak kinetochore that interacts less robustly with the microtubule spindle. (**A**) Maximum intensity projection of a male zygote stained for the kinetochore protein Hec1 (cyan). (**B**) Quantification of normalized Hec1 fluorescence intensity of individual centromeres from CBAxB6 F1 parents. Each dot represents a single centromere of a male zygote. Kinetochores analyzed: *n* = 1891 autosomes; 34 chrX; 49 chrY. (**C**) Scheme illustrating how the absence of canonical major satellite repeats in the centromeric region distinguishes chrY from all other mouse chromosomes, and allows for identification of chrY in live/fixed zygotes in a way that does not bias its behavior. (**D**) DNA FISH of the male zygote shown in (E). (**E**) Maximum intensity projection of a male zygote microinjected with mRNA encoding MajSatTALE-StayGold protein (green), followed by immunofluorescence labeling for CENP-A (yellow). Insets show the labeling patterns for the chrX and chrY. ChrY can be distinguished among the 20 mouse chromosomes by the lack of fluorescent signal (green). (**F**) Scheme illustrating the experimental approach used to assess microtubule-induced stretch on chrY in 3D preserved spindles from zygotes. (**G**) Representative maximum intensity projection of a male zygote treated as in (F). (**H**) Quantification of the iKT at chrY (unlabeled) versus other chromosomes (labeled) in zygotes from C57BL/6J mice. Each dot represents a single chrY (right) or the mean for all the other chromosomes in the cell (left). *n* = 15 zygotes. Boxes mark regions that are magnified in insets. Arrowheads highlight individual centromeres of sex chromosomes. Scale bars, overviews (10 μm); insets (1 μm). Statistical significance was evaluated using Kruskal-Wallis test (B) and Wilcoxon matched-pairs signed rank test (H). Error bars show means ± SD.

To examine chromosome behavior in spindles reconstructed in three dimensions (confocal microscopy, fixed cells; Fig. 3, C to H) and in live cells [high–temporal resolution light-sheet microscopy (*50*) (Fig. 4, A to H)], we searched for a probe that uniquely distinguishes the Y but does not bias its segregation behavior. To achieve this, we used a probe that labels all chromosomes except the Y (fig. S5, C and D) by leveraging the unique lack of canonical major satellite DNA repeats in the pericentromeric region of the Y (Fig. 3C) (*51*). We expressed mRNA encoding a transcription activator–Like (TAL) effector protein targeting these major satellite repeats and a fluorescent reporter (MajSatTALE-StayGold). This approach has been previously shown to be compatible with studying embryonic divisions (*52*). We confirmed that after MajSatTALE-StayGold mRNA expression, all chromosomes in female zygotes were labeled (fig. S5, D and E), while in males, exactly one chromosome was consistently unlabeled (Fig. 3E and fig. S5C). The unlabeled chromosome was always the Y, which was further confirmed by DNA FISH, thus validating the specificity and sensitivity of our approach (Fig. 3, D and E, and fig. S5F). We then fixed zygotes expressing MajSatTALE-StayGold at the first metaphase and labeled their centromeres (CENP-A, yellow) and microtubules (α-tubulin, white) in three dimensions (Fig. 3, F and G, and movie S3). The Y chromosome’s two sister centromeres had a smaller inter-kinetochore distance (iKT), indicating that the Y chromosome is under less tension from spindle microtubules (Fig. 3, G and H). This effect was directly dependent on bipolar microtubule pulling as monastrol treatment that prevented chromosome biorientation eliminated the difference in iKT between the Y chromosome and the X chromosome or autosomes (fig. S5, G and H).

**Fig. 4.**
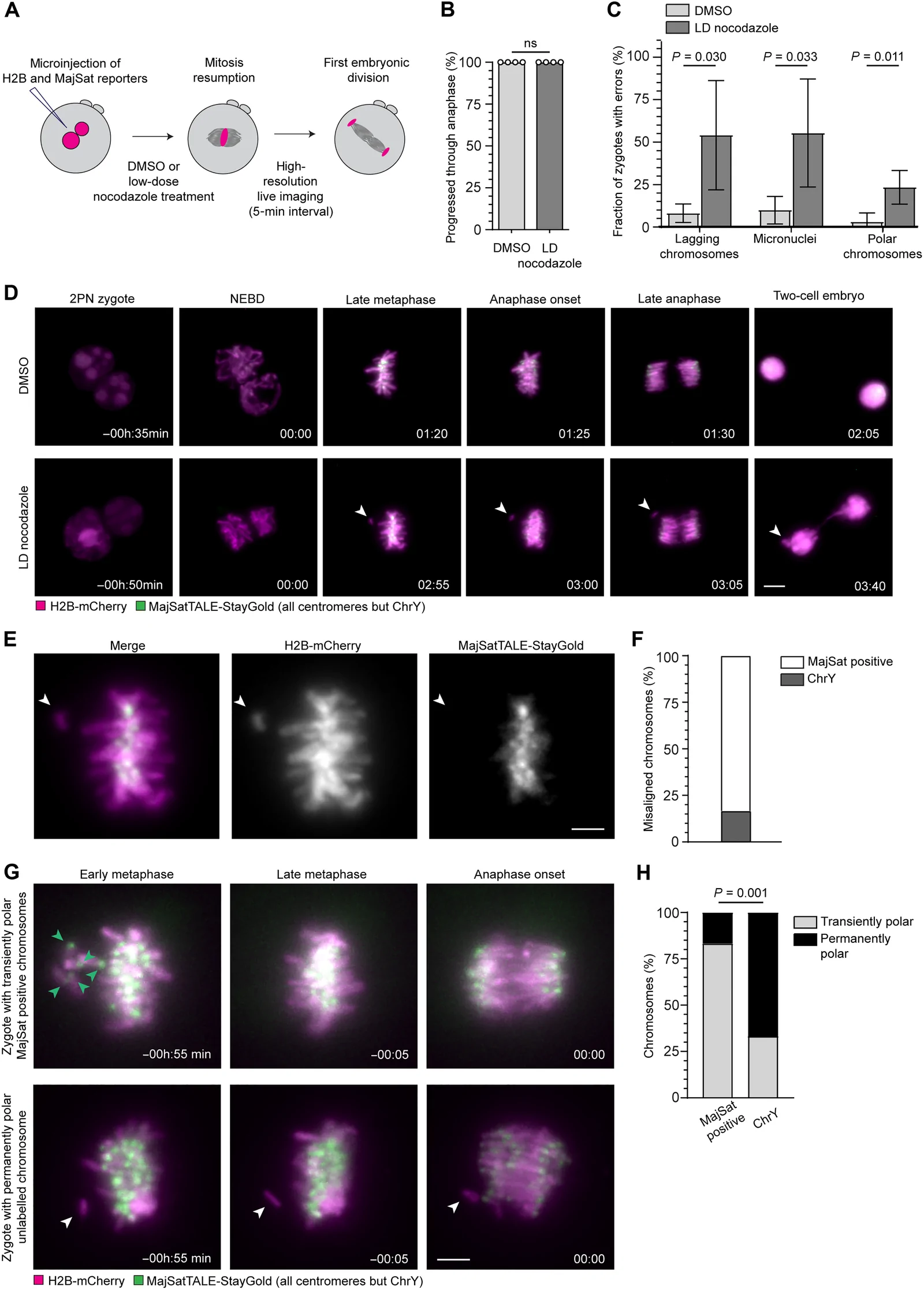
The Y chromosome is prone to misalignment and frequently fails to realign at the pole before anaphase onset. (**A**) Experimental approach to study the first mitotic division in live zygotes. Upon expression of MajSat probe, only one chromosome in male zygotes is unlabeled (see also fig. S5, C and D). (**B**) Quantification of the fraction of zygotes, which progress through anaphase under control conditions [dimethyl sulfoxide (DMSO)] and low-dose (LD) nocodazole conditions. *N* = 4 experimental repetitions, *n* = 63 control group and *n* = 87 experimental group zygotes. (**C**) Quantification of the fraction of zygotes as in (B), which progress through anaphase with errors. (**D**) Live zygotes imaged at a temporal resolution of 5 min. Top, control (DMSO); bottom, low-dose nocodazole. (**E**) Magnified maximum projection image of the zygote from (D) at one time frame before anaphase onset. (**F**) Quantification of misaligned chromosomes (*n* = 72 chromosomes) from the four experimental repetitions as in (B), based on the presence or absence of MajSat probe. (**G**) Representative images of labeled misaligned chromosomes that return to the spindle equator before anaphase onset (top, transiently polar), and an unlabeled chrY that permanently remains at the pole (bottom). (**H**) Quantification of the fraction of MajSat-positive versus MajSat-negative (chrY) chromosomes as in (F) that either permanently remain at the spindle pole or transiently move to the pole but then return to the spindle equator before anaphase onset. White arrowheads point toward chrY; green arrowhead point toward MajSat-positive polar chromosomes. Scale bars, 10 μm (D) and 5 μm (E and G). Error bars show means ± SD. Statistical significance was evaluated using Mann-Whitney test (B), *t* test (C), and Fisher exact test (H).

Prolonged microtubule pulling can lead to cohesion fatigue, where the two sister chromatids prematurely fall apart and can generate complex aneuploid phenotypes as seen in cancer cells (*53*–*55*). Previous work showed that predisposition to cohesion fatigue across human chromosomes is nonrandom (*55*). To test whether the Y that is under less tension is also less prone to cohesion fatigue, we exposed embryos to different durations of microtubule pulling (∼3 versus ∼15 hours; fig. S6A). While cohesion fatigue is common in somatic cells (>80% by 8 hours) (*53*), metaphase-II arrested oocytes (*56*), and more advanced embryos (*57*), 15 hours of pulling unexpectedly did not induce any chromosomes disintegrating into single chromatids in any of the zygotes studied (fig. S6B). However, prolonged microtubule pulling damaged chromosome integrity and increased the iKT distance (fig. S6C; baseline: 1.67 μm, after 12 additional hours: 2.99 μm). Our analysis revealed that the Y chromosome is less prone to cohesion fatigue than the X (fig. S6, D and E). Intriguingly, this resilience was not a general feature of paternal chromosomes, which were slightly but significantly more susceptible to cohesion fatigue than maternal chromosomes (fig. S6, F and G). In addition, the resistance to cohesion fatigue was not a consequence of a higher total cohesin load as levels of the cohesin subunit Smc3 were lower on the Y chromosome than on the X or autosomes (fig. S7, A and B). In summary, these findings uncover additional asymmetries between parental sex chromosomes, showing that the weak Y centromere forms a weaker kinetochore and interacts less robustly with spindle microtubules. While the weak kinetochore may contribute to the resistance against cohesion fatigue, relying on fewer microtubule bundles could sensitize the Y chromosome to erroneous kinetochore-microtubule attachments.

### The Y chromosome often fails to align at the spindle equator and is prone to being trapped at the spindle pole

Our observation that the Y chromosome interacts less strongly with the spindle raised the possibility that it is particularly vulnerable to even mild perturbations in microtubule dynamics. To test this idea, we monitored the first embryonic mitosis at a high temporal resolution (light-sheet microscopy, 5-min intervals) in the presence or absence of low-dose nocodazole (*57*), which partially depolymerizes spindle microtubules (Fig. 4A). Since microtubules are critical for unifying the two pronuclei before the first mitosis (*43*), we added nocodazole only after pronuclear migration was completed (fig. S8, A and B). We identified a nocodazole concentration that generated the expected prolongation of mitosis (249 versus 87 min; fig. S8C) but did not affect the efficacy or timing of nuclear envelope breakdown (fig. S8, D and E) and allowed 100% of zygotes to proceed through anaphase (Fig. 4B). Consistent with successful partial depolymerization, the incidence of lagging chromosomes (51.0%), micronuclei (52.2%), and misaligned chromosomes at the pole (21.8%) was increased (Fig. 4C).

High-resolution time-lapse imaging revealed that the Y chromosome preferentially misaligned toward the spindle pole (Fig. 4, D and E, and fig. S8F; Y marked with an arrowhead). Assuming equal probabilities, we would expect 1.25% of polar chromosomes to be TALE-negative (1 in 40 chromosomes being Y and ∼50% of zygotes being male). However, the actual rate was 16.7% (Fig. 4F). Moreover, while TALE-positive chromosomes returned to the spindle equator before anaphase in 83.3% of cases (movie S4, green arrows), the Y chromosome returned in only 33.3% of cases (movie S5, white arrow, and Fig. 4, G and H, “transiently polar”). Given that we showed that Y’s Hec1 levels are low (Fig. 3B), this may reduce its ability to effectively glide (*58*) along lateral microtubules and impair its ability to realign at the spindle equator. In summary, our results indicate that the Y chromosome is more prone to misalignment and less likely to be recaptured from the spindle pole before anaphase onset.

### Y chromosome loss compromises embryo growth, but Y-lacking cells are partially counterselected during preimplantation development

Our data identified centromere vulnerability of the Y that could put the embryo at risk of aneuploidy. This raised an intriguing question, namely, what is the fate of cells with this missegregation? A chromosome missegregation event in the zygote would result in all future embryo cells inheriting the aneuploidy (fig. S9A, middle row), while a missegregation in one blastomere at the two-cell stage could produce a mosaic 50:50% euploid:aneuploid four-cell stage embryo with an unknown developmental potential (fig. S9A, bottom row). Previous studies have shown that mouse and human mosaic embryos can deplete cells with complex aneuploidies from the late blastocyst stage onward (*59*–*61*). However, in those studies, aneuploidy was not specific to a given chromosome and involved multiple chromosomes simultaneously (*59*–*61*). Here, we sought to determine whether embryos also eliminate blastomeres that have specifically lost the Y chromosome.

To achieve this, we created a label-free system to generate embryo mosaics containing two euploid XY blastomeres and two XO blastomeres that lacked the Y chromosome (Fig. 5A). XO embryos were produced by crossing wild-type females with males that produce “O” gametes (fig. S9B) (*62*). Using both blastomeres from each embryo for aggregation was crucial as the two blastomeres at the two-cell stage might differ in their developmental potential (*63*, *64*), which could bias the results. We cultured the aggregates to the late blastocyst stage and then fixed them and identified the sex chromosome complement of the two donors using DNA FISH. Aggregation was highly efficient, with 95.4% of control aggregates developing into a unified blastocyst (fig. S9, C and D). Control XX-XY aggregates showed an equal contribution from the two donors (fig. S9E, 48.93% XX:51.07% XY), confirming that aggregation did not harm cell viability and that both XX and XY cells can contribute equally to the developing blastocyst.

**Fig. 5.**
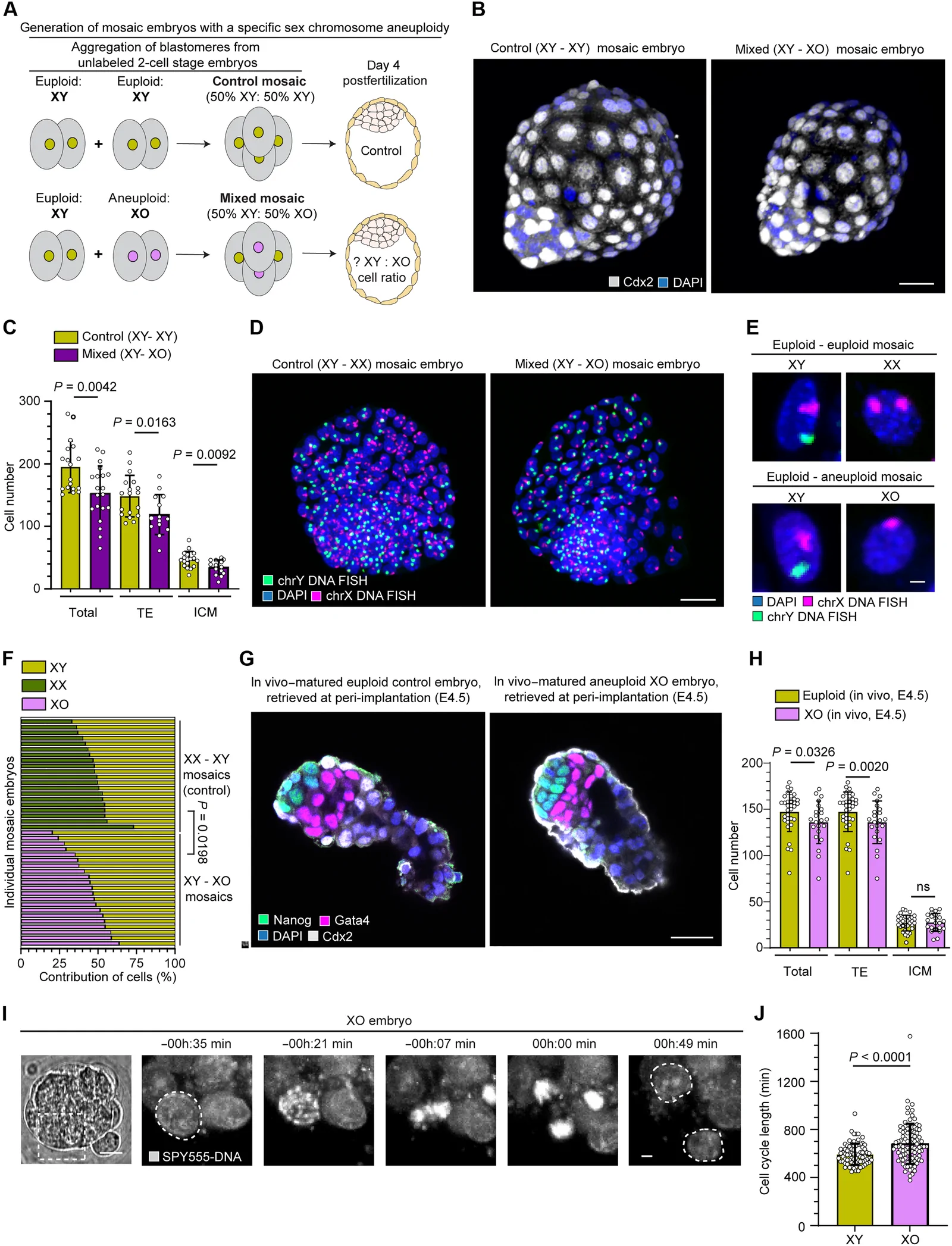
Embryo plasticity allows euploid XY cells to partially outcompete XO cells, reducing the fitness cost of Y chromosome missegregation. (**A**) Blastomere aggregation procedure for generating mosaic embryos. (**B**) Representative control (XY-XY) and mixed mosaic (XY-XO) embryos from the same experiment. (**C**) Quantification of the number of cells in control (XY-XY) and mixed (XY-XO) mosaics (*N* = 17 experiments). Cells were split into TE and ICM based on Cdx2 labeling as in (B). Analyzed: 18 control and 20 mixed aggregates for total cell number, and 18 control and 14 mixed aggregates for TE/ICM composition. (**D**) Maximum intensity projection of mosaic embryos from the control (XX-XY) and mixed (XY-XO) groups. (**E**) Cell types identified by DNA FISH in control (XX-XY) and mixed (XY-XO) mosaics, as shown in (D). (**F**) Relative contribution of the XY and XO clones in control and mixed mosaics, analyzed as in (E). (**G**) Representative euploid control and nonmosaic XO embryos developed in vivo and collected periimplantation at E4.5. (**H**) Quantification of cell number from in vivo–matured embryos, as in (G). Analyzed: 33 control and 22 XO embryos from seven experiments. (**I**) Representative live-imaging snapshots of an XO embryo during the fifth cleavage. Times are relative to anaphase onset. (**J**) Duration of cell cycle for XY versus XO blastomeres during the fifth cleavage, imaged as in (I). Analyzed: 78 XY and 98 XO blastomeres from four mice across two independent experiments. Scale bars, 30 μm (B, D, and G); 3 μm (E); 20 μm in overviews and 4 μm in insets of (I). In all panels, the control and experimental images are to scale. Statistical significance was evaluated using Welch’s *t* test (C and J), unpaired *t* test (F), and Mann-Whitney test (H). Error bars show means ±. SD.

We then investigated whether losing the Y chromosome affects embryo development by comparing XY-XY and XY-XO mosaics. XY-XO mosaics were on average smaller than XY-XY controls (Fig. 5, B and C, and movie S6), a trend consistent across total cell number (153 in XY-XO versus 195 in XY-XY), trophectoderm cells (TE, 119 versus 148 cells), and inner cell mass (ICM, 35 versus 47 cells). These data show that having half of the cells aneuploid for a single sex chromosome is enough to reduce embryo size by the late blastocyst stage. To explore whether aneuploid cells were outnumbered by euploid cells, we quantified the contribution of each clone in XY-XX and XY-XO mosaics using DNA FISH (Fig. 5, D and E, and fig. S9F). While control aggregates contained nearly equal 50:50% contributions, XO cells were less prevalent in mixed aggregates, making up only 43.9% of the cells (Fig. 5F).

We next asked whether these findings reflect intrinsic properties of XO cells or arise only when these aneuploid cells coexist with euploid counterparts. To address this, we assessed embryo size in nonmosaic embryos developed in vivo to the periimplantation stage. Under these conditions, XO embryos were smaller and contained fewer TE cells than euploid controls (Fig. 5, G and H). To determine how this size difference arises, we tracked cell cycle progression during a single division (fifth cleavage) in nonmosaic embryos using light-sheet microscopy (Fig. 5I). This analysis revealed a prolonged cell cycle in XO compared to XY blastomeres (680.6 versus 592.8 min; Fig. 5J). Together, these findings indicate that loss of a single sex chromosome is sufficient to affect mitotic proliferation, while developmental plasticity can partially compensate for Y chromosome loss during preimplantation development in mosaic embryos.

## DISCUSSION

In this study, we find that in the zygote, the Y chromosome exhibits unusually low levels of CENP-A. This cannot be explained by the diminished satellite content on the Y because CENP-A levels increase during subsequent embryonic mitoses. While most unperturbed embryos manage to segregate the Y chromosome, we show that even mild experimental challenges can drive its missegregation through polar entrapment. The predisposition of the Y chromosome to polar fate that we identify is likely of less significance to somatic cells or spermatogenesis because their spindle assembly checkpoint (SAC) mechanisms are robust and delay anaphase onset until all chromosomes reach alignment (*65*). In contrast, early mammalian embryos are well known for a weak SAC [see Fig. 4B and (*57*)], which explains why the peculiar properties of the Y chromosome are a particular threat to early embryos. The vulnerability that we have identified may pose a risk to sex determination, which in mammals relies on the presence or absence of the Y (*19*–*21*). It may also represent a previously unrecognized etiological factor contributing to Turner syndrome. It would be interesting to determine the incidence of Y chromosome loss in unperturbed development and to identify physiological stressors that impair CENP-A incorporation in embryos and thus further exacerbate Y chromosome loss. Our data suggest the Y chromosome copes well with the reduced CENP-A levels in the aged zygote caused by female reproductive senescence, aligning with large population studies that find no impact of maternal age on the likelihood of the offspring being male (*66*).

Our insights into the Y chromosome’s strengths and vulnerabilities may also have broader implications for adult health. We demonstrate chromosome-specific differences in Shugoshin2 and Smc3 levels, alongside a relative resistance of the Y to cohesion fatigue. Although a recent study identified that porcine autosomal acrocentric chromosomes in oocytes exhibit lower cohesin levels than metacentrics (*67*), these chromosomes are more prone to cohesion fatigue, raising the possibility that the Shugoshin2-mediated compensation observed for the mouse zygotic Y is absent in that context. The reduced susceptibility of the Y chromosome to cohesion fatigue, despite its lower cohesin load, may reflect compensatory features such as enhanced cohesion protection and a smaller kinetochore that exposes the chromosome to reduced microtubule forces. Moreover, this study detects a discrepancy in CENP-A between sister Y centromeres in the zygote, which may result from limiting loading factors, replication timing, or transient developmental-stage–specific differences in chromatin accessibility. Given that the Y centromere is resilient to a physiological reduction in the total CENP-A level pool brought about by aging, this narrows down the likely driver of Y chromosome missegregation in aging somatic cells. Our results point toward intrinsic age-related changes in Y centromere architecture or in CENP-A deposition machinery as interesting areas for further study in the context of Y chromosome loss and increased disease burden as men age.

In addition, this work supports the notion of inherent plasticity during early embryogenesis that favors embryo euploidy and intergenerational fitness. We identify two mechanisms by which Y chromosome loss influences preimplantation development: XY-XO mosaics are smaller in size, and XY cells can outcompete XO cells within individual embryos. The increase in cell cycle length in XO versus XY blastomeres observed in our study aligns with the decreased proliferation of XO human fibroblasts and relative reduction in XO fraction when these somatic cells are cocultured with euploid counterparts (*68*, *69*). In line with these findings, individuals with mosaic Turner syndrome also exhibit a progressive, age-related depletion of XO cells (*70*). In mice, where blastocyst number exceeds available implantation sites, this size reduction may reduce the likelihood of less advanced mosaic blastocysts implanting, potentially mitigating the consequences of transient Y instability without affecting litter size. In addition, the competitive advantage of XY cells over XO cells may help ensure that surviving embryos contain minimal aneuploid cells, reducing the long-term effects of early Y loss on adult health. An alternative hypothesis is that XO cells may be preferentially relegated to extraembryonic tissues after implantation, thereby mitigating their physiological impact in mosaic embryos. This possibility warrants further investigation, given case reports of confined XO placental monosomy in euploid newborns (*71*) or XO placental monosomy in XY-XO mosaic fetuses (*72*).

The counterselection of XO cells is also interesting from a broader perspective, as while aneuploid cells have previously been shown to be eliminated from mosaic mouse periimplantation embryos (*59*, *60*) and human gastruloids (*61*), these studies relied on pharmacological perturbations to generate aneuploid cells, resulting in complex and unpredictable aneuploidies affecting multiple chromosomes simultaneously. Our work shows that mildly aneuploid cells, where a single specific chromosome in preimplantation embryos is affected, can be outcompeted during early embryonic development.

Last, it is intriguing that Y chromosome absence can influence preimplantation development given that it is gene-poor with few known roles in early embryogenesis (*21*). However, several limitations of this study should be considered when interpreting the findings and their broader implications. First, the molecular basis for the particular vulnerability of the Y chromosome to changes in CENP-A during the gamete-to-zygote transition remains unclear. Future studies should therefore address how CENP-A levels are reestablished during preimplantation development and why this process may affect the Y chromosome disproportionately. Second, this study focused specifically on the consequences of Y chromosome loss for embryonic development. It therefore remains uncertain to what extent the effects on preimplantation developmental trajectory identified here are generalizable to the loss of other individual chromosomes. Moreover, further work will be required to determine whether the observed reduction in blastomere proliferation reflects a general consequence of chromosomal instability or results specifically from the loss of Y-linked genes. These unresolved questions are particularly relevant in the context of human preimplantation development as the first zygotic division in humans is associated with even more challenges than in mice, including spindle instability (*73*, *74*), dependence on decades-old maternal factors (*43*, *75*), and replication stress (*76*). It would therefore be very interesting for future studies to uncover how the human Y centromere copes with the unique challenges specific to the beginning of life.

## MATERIALS AND METHODS

### Animals—Mouse strains and mating schemes

All animal research was conducted in accordance to the United Kingdom Animal (Scientific Procedures) Act 1986, approved by institutional Animal Welfare and Ethical Review Body (the Francis Crick Institute PPL Review Committee) and conducted under authority of the Home Office approved Project Licence PP1234304. Mice were provided ad libitum access to food and water and housed in individually ventilated cages maintained at 22°C with 60% humidity on a 12-hour light-dark cycle. The mouse strains used included C57BL/6J [wild type, XY^*Sry*−^ (*40*) and XY^*Sry*−^/Tg(Sry) (*41*), F1 hybrid (C57BL/6J x CBA) and MF1 (wild type and X^Y^ (*62*)]. XY^*Sry*−^ males were used to study the properties of the Y chromosome in the context of female oocyte meiosis (Fig. 1 and fig. S3); XY^*Sry*−^/Tg(Sry) males were used to confirm that the Y centromere properties in XY^*Sry*−^ mice are not merely a nonspecific off-target consequence of Sry gene deletion (fig. S3); X^Y^ males were used to generate XO mosaic and nonmosaic embryos (Fig. 5 and fig. S9). For comparisons of maternal age (fig. S4), F1 hybrid (C57BL/6J × CBA) females aged 6 to 9 weeks (young) or 52 to 65 weeks (old) were used. For wild-type matings, embryos were generated using males and females of the same strain and genotype.

C57BL/6J and MF1 mice were typically naturally mated, whereas F1 hybrid (C57BL/6J × CBA) females were primarily superovulated before mating (except for the young and aged comparisons in fig. S4). Superovulation was deemed necessary because natural mating routinely produces only about eight embryos, and this study focused on sex-specific phenotypes, yielding an average of four embryos of the desired sex per female. Thus to obtain larger embryo numbers, superovulation was used in accordance with the 3Rs principles of animal welfare. Because of the low superovulation efficiency in the C57BL/6J strain, experiments requiring a large number of zygotes were mainly performed using the F1 hybrid (C57BL/6J × CBA) parents. These females were superovulated by injecting 5 IU of pregnant mare serum gonadotropin (Sigma-Aldrich) between 12 p.m. and 2 p.m. Forty-eight hours later, 5 IU of human chorionic gonadotropin (Sigma-Aldrich) was administered, and the mice were paired with wild-type F1 hybrid strain (C57BL/6J × CBA) males. Vaginal plugs were checked the following morning to confirm successful mating.

### Embryo isolation and culture

Embryo isolation was performed using handling medium (FHM, prepared in-house). Zygotes, two-cell stage and four-cell stage embryos were obtained by gentle oviduct puncture, while eight-cell embryos and blastocysts were collected by flushing the oviduct and uterus, respectively. For zygotes, cumulus cells were removed by gentle pipetting in FHM medium containing hyaluronidase (Sigma-Aldrich, H4272). After washing, embryos were transferred to preequilibrated drops of KSOM medium under mineral oil (Origio, ART-4008-5P) and cultured at 37°C with 5% CO_2_ until further experimental steps.

### 2D metaphase chromosome spreads and immunostaining in embryos and oocytes

Embryos at the desired developmental stages were synchronized at metaphase by incubating them in KSOM medium supplemented with 100 μM APCin [diluted in dimethyl sulfoxide (DMSO), Tocris Bioscience, 5747], as described previously (*34*). For cohesion fatigue experiments (fig. S6), zygotes from individual mice were divided into two groups: One was processed at 10 p.m. on the day a vaginal plug was detected, and the other ∼2 hours later. Two-cell and 8-cell stage embryos were fixed between 4 p.m. and 6 p.m. on embryonic days 1 and 2 (E1 and E2), respectively, while four-cell stage embryos were processed between 6 a.m. and 8 a.m. on E1. Strict timing was essential because of the increased sensitivity to cohesion fatigue in embryos beyond the zygotic stage. For meiosis I oocyte spreads, oocytes were collected in M2 medium (Sigma-Aldrich, M7167) and incubated at 37°C for 5 to 7 hours.

The chromosome spreading protocol was adapted on the basis of the previously described protocol for mouse oocytes (*77*). At the start of the spreading protocol, the zona pellucida was removed by briefly washing the embryos in Tyrode’s solution (Sigma-Aldrich, T1788) at room temperature under mineral oil. Embryos were then quickly washed through KSOM droplets (or M2 medium for oocytes) to eliminate any residual Tyrode’s and placed on an agar plate containing a 1:1 fetal bovine serum solution at 37°C for exactly 14 min.

To spread chromosomes, embryos were gently transferred into a 7-μl drop of spreading solution on a 15-well multitest slide (MP Biomedicals, 096041505). The spreading solution was prepared freshly on the day and consisted of 1% (v/v) paraformaldehyde (pH 9.2 to 9.4; dissolved at 65°C from frozen stock and then cooled down at room temperature for at least 30 min before initiating the protocol), 0.15% (v/v) Triton X-100 and 3 mM dithiothreitol. Care was taken to minimize the volume of solution transferred. The slides were placed in a closed humidified chamber for at least 3 hours.

Afterward, the lid of the chamber was opened at a 45° angle, and the slides were air-dried. To prevent overdrying, which may compromise antibody labeling quality, the slides were air-dried for no more than 3 hours before being stored at −80°C until further use.

On the day of labeling, slides were thawed and washed in phosphate-buffered saline (PBS), followed by immunowash solution, as previously described (*77*). When using the CENP-A antibody, slides were incubated with 5 μl per well of λ-phosphatase (New England Biolabs, P0753S) for 2 hours at 30°C in a hybridization oven, as described previously (*28*, *44*). Immunostaining was carried out by sequential incubation with primary antibodies (overnight at 4°C or 3 hours at room temperature) and secondary antibodies (2 to 3 hours at room temperature) in a humidified chamber. During all incubation steps, slides were covered with a large coverslip to prevent evaporation.

Primary antibodies used on spreads were as follows: CENP-A (1:200 dilution; #2048S, Cell Signaling Technology—all figure panels apart from fig. S2 (C, D, and F), where an alternative CENP-A antibody was used to independently verify the findings—1:200 dilution; #MA5-14829, Invitrogen); Hec1 (1:50 dilution; clone C-11 sc-51550, Santa Cruz Biotechnology); H3K9me3 (1:100 dilution; 39285, Active Motiff); SGOL2 (1:200 dilution; orb499806, Biorbyt), and Smc3 (1:400 dilution; ab9263, Abcam). Secondary Antibodies were Alexa Fluor 488/594/647–labeled (Invitrogen), used at 1:100 to 1:400 dilution. DAPI (4′,6-diamidino-2-phenylindole) was used at 5 μg/ml (D9564-10mg Sigma-Aldrich). Before microscopy, slides were gently dried and covered with 2 μl of VECTASHIELD Antifade Mounting Medium (2B Scientific, H-1000) per well, followed by mounting with a 22 mm–by–50 mm glass coverslip of #1.5 thickness (797582, Menzer-Glaser) and sealing with nail varnish. After confocal microscopy imaging, the slides were stored at 4°C until FISH protocol was initiated.

### 2D chromosome painting (DNA FISH) on embryonic or oocyte metaphase spreads

Following immunolabeling and confocal imaging (see above), the slides were subjected to a DNA FISH protocol based on a previously described method (*78*). Coverslip slides were washed twice in PBS (5 min each) and then passed through a dehydration ethanol series: 70% ethanol (2 × 2 min), 90% ethanol (2 × 2 min), and 100% ethanol (5 min). The slides were subsequently washed three times in 2× SSC (pH 7 to 7.5) and denatured in 2× SSC at 80°C for 5 min. Immediately after denaturation, the slides were quenched in ice-cold 70% ethanol and subjected to a second ethanol dehydration series as described above. The slides were then gently air-dried, and 2 μl of premixed DNA FISH probe solution (XMP Y Orange D-1421-050-OR and XMP X Green D-1420-050-FI, Metasystems) was added to each well. In some experiments, the fluorophores were used in the reverse combination. A large coverslip (22 mm by 64 mm) was placed over the slide, which was incubated on a 75°C heat block for 5 min. The slide was then transferred to a humidified chamber and incubated at 37°C overnight.

The next day, the coverslip was gently removed by briefly washing the slide in room-temperature 2× SSC. The slide was then placed in 0.4× SSC for 2 min at 72°C, followed by a wash in 2× SSC with 0.05% Tween-20 for 2 min at room temperature. After rinsing with distilled water, the slide was incubated with DAPI (D9564, Sigma-Aldrich) for 10 min. Following another distilled water rinse, the slide was gently dried and mounted as described previously. The slide was then subjected to a second round of confocal imaging. Chromosome morphology and xyz coordinates were used to match the immunolabeled images with the DNA FISH images. In the few experiments where the sensitivity and the specificity of the MajSatTALE-StayGold labeling approach was confirmed, immunolabeling and DNA FISH were preceded with microinjection with MajSatTALE-StayGold mRNA (see below).

### ESCs: 2D metaphase chromosome spreads and immunolabeling

The protocol for metaphase chromosome spreads was adapted from previously published methods (*55*, *79*). Mouse ESCs were cultured in 2i/LIF media as previously described (*80*). ESCs were split and cultured for 24 hours before treatment with 1% colcemid for 3 hours at 37°C with 5% CO_2_. Metaphase-arrested cells were collected in Dulbecco’s PBS (DPBS) by mitotic shake-off and washed in DPBS. The cells were resuspended in 75 mM KCl hypotonic solution for 10 min at 37°C, followed by incubation for 10 min on ice. Then, ESCs were transferred to cytospin cones with adhesion slides (Superfrost Plus Adhesion slides, Epredia) and centrifuged at 1800 RPM for 10 min. The slides with spreads were fixed in cold and freshly prepared PTEMF fixative (0.2% Triton X-100, 0.02 M Pipes at pH 6.8, 0.01 M EGTA, 1 mM MgCl_2_, and 4% formaldehyde) for 10 min and then washed three times in PBS with 0.1% Triton X-100. Before immunolabeling, the metaphase spreads were incubated with λ-phosphatase (New England Biolabs, P0753S) for 1 hour at 30°C as described previously (*28*, *44*) and then blocked for 30 min with 3% bovine serum albumin (BSA, Sigma-Aldrich, 9647) in PBS with 0.1% Tween-20. Immunostaining followed with sequential incubation with primary antibody CENP-A (1:200 dilution; Cell Signaling Technology, 2048S) overnight at 4°C and secondary antibody Alexa Fluor 488–conjugated (1:400 dilution; Invitrogen) together with DAPI (1:500 dilution; Sigma-Aldrich, D9564) for 2 hours at room temperature covered with a coverslip and in a humidified chamber. Coverslips were mounted using Vectashield antifade mounting medium (Vector Laboratories, H-1000).

### In vitro mRNA synthesis, zygote microinjection

Chromosomes were labeled using previously described H2B-mRFP mRNA (*67*). To label the major satellite sequences of mouse centromeres, the 15–base pair published sequence (TGCCATATTCCACGT) (*52*) was targeted with a TALE. Fluorescent TALE construct was created in house as per previously published protocol using a two-step Golden Gate assembly (*81*), with the RVD plasmids obtained from Addgene (Golden Gate TALEN and TAL Effector Kit 2.0, #1000000024). However, in the destination vector (pTALYM3, Addgene, #47874), the mClover fluorescence protein was exchanged with StayGold (subcloned from Addgene plasmid #186296) to achieve a more optimal fluorescent signal intensity for live imaging. H2B-mRFP was linearized with AscI, while MajSatTALE-StayGold with PacI. All mRNAs in this study were synthesized using a T7 polymerase (mMessage mMachine kit, Ambion) according to the manufacturer’s protocol.

Zygotes were transferred to microdrops of FHM medium covered with mineral oil in glass-bottom dishes (35-mm dish with 14-mm well, #1.5 glass; D35-14-1.5-N, Cellvis), which had in-house heat-melted edges to increase the working distance. Microinjection was performed using the Eppendorf FemtoJet 4i with the following parameters: injection pressure 300 to 400 hPa, compensation pressure 20 hPa. The microinjection mix consisted of MajSatTALE-StayGold only (Fig. 4 and fig. S6) or a premixed solution of H2B-mRFP and MajSatTALE-StayGold (for live imaging experiments). Injection needles were prepared in-house using TW100F-6 (World Precision Instruments) and bent with a microforge, while the holding capillary was MPH-MED-30 (Cooper Surgical). After injection, zygotes were transferred to preequilibrated KSOM at 37°C with 5% CO_2_, awaiting further experimental steps.

### SPY dye labeling in zygotes

In some experiments (e.g., fig. S1D), zygotes were labeled using SPY dyes (Spirochrome) using conditions previously shown to be compatible with unperturbed preimplantation development (*82*). The zona pellucida was removed with Tyrode’s solution (Sigma-Aldrich, T1788) before dye addition as this greatly enhanced labeling strength at the zygote stage. Briefly, zygotes were placed in a 500-μl solution of SPY555-DNA (diluted to 1:1,000) and SPY620-Actin (diluted to 1:2,000) in KSOM for 1.5 hours at 37°C with 5% CO_2_. This incubation was done without an oil overlay to improve labeling consistency. Afterward, the zygotes were washed into a mix of SPY555-DNA and SPY620-Actin (each at 1:1,500) and either control (DMSO at 1:1,000) or APCin (100 mM stock in DMSO, diluted at 1:1,000 in KSOM; Tocris Bioscience, 5747). The zygotes were then transferred in groups to the light-sheet holder containing the same solutions, covered with mineral oil (Origio, ART-4008-5P).

### Spindle perturbations in zygotes using low-dose nocodazole and monastrol

The selected dose of nocodazole for partial depolymerization of microtubules was 15 to 25 nM, as a titration analysis showed that at this concentration, the vast majority of embryos progressed through anaphase despite challenges to chromosome segregation. Embryos were microinjected as described above and allowed to express the mRNA for 2 to 3 hours. Subsequently, they were washed into either nocodazole (stock 10 mM, diluted to 10 μM in DMSO and further diluted to the desired concentration in KSOM) or DMSO (control; dilution matched to the experimental group). The embryos were then transferred in groups to small indentations within the light-sheet holder and imaged in group culture (control or nocodazole) overnight.

To generate monopolar spindles with monotelic kinetochore–microtubule attachments, zygotes were retrieved on day 1 after a positive plug check. Following retrieval, zygotes were split into two groups and cultured in KSOM medium supplemented as following: control (APCin, 100 μM; Tocris Bioscience) and experimental (APCin, 100 μM, and monastrol, 0.1 mM; Sigma-Aldrich, M8515). DMSO concentrations were adjusted to match between control and experimental groups. The following morning, embryos were subjected to chromosome spreading, followed by immunofluorescence for CENP-A (#2048S, Cell Signaling Technology) and DNA FISH for the X and Y chromosomes (see above).

### Immunolabeling in fixed zygotes and periimplantation embryos with preserved 3D architecture

Zygotes were injected with MajSatTALE-StayGold mRNA as described above and arrested at metaphase in KSOM medium supplemented with APCin (100 μM, Tocris Bioscience, 5747). Subsequently, cells were exposed to a 15-min cold treatment to partially depolymerize nonkinetochore-bound microtubules. Cells were then fixed in 100 mM Hepes, 10 mM MgSO_4_, 50 mM EGTA, 0.5% Triton X-100 (v/v), and 2% (v/v) formaldehyde for 30 min at 37°C, followed by blocking overnight at 4°C in PBS containing 0.3% (v/v) Triton X-100, as described previously (*67*). The following day, lambda phosphatase treatment was performed, as described above. Cells were blocked with 3% (w/v) BSA (Sigma-Aldrich, 9647) in PBT for 30 min. Primary antibodies were added sequentially: CENP-A (1:200 dilution; 2048S, Cell Signaling Technology) and α-tubulin (Bio-Rad, MCA78G). Secondary antibodies used were Alexa Fluor 594 anti-rabbit and Alexa Fluor 647 anti-rat. DAPI was used at 5 μg/ml (D9564, Sigma-Aldrich) to stain the DNA.

To determine embryo size in nonmosaic in vivo matured periimplantation embryos, wild-type or X^Y^ males were mated with wild-type females. The females were then euthanized on day 4.5 after a positive plug check (E4.5). Embryos were isolated from the uteri, as described above. Immediately after retrieval, embryos were fixed and subject to an immunolabeling protocol (see above). The following antibodies were used: Cdx2 (1:200 dilution; R&D Systems, AF3665-SP), Gata4 (1:500 dilution; Thermo Fisher Scientific/eBioEvan, 14-9980-80) and Nanog (1:500 dilution; Cosmo bio, RCAB001P). The labeling pattern and cell position were used to count the nuclei and allocate them to the respective lineages. Secondary antibodies used were Alexa Fluor 488 anti-rabbit, Alexa Fluor 594 anti-goat, and Alexa Fluor 647 anti-rat. DAPI was used at 5 μg/ml (D9564, Sigma-Aldrich) to stain the DNA. After a round of confocal imaging, the embryos were subject to a DNA FISH protocol and another round of imaging, as described also below.

### Generation and processing of mosaic blastocysts with sex chromosome aneuploid cells

Wild-type MF1 female mice were mated either with wild-type males of the same strain or with X^Y^ males, the latter of which generate sex chromosome aneuploid X^y^ or “O” gametes. Embryos from these matings were retrieved at the two-cell stage at midday of E1.5. The zona pellucida was removed by a brief exposure to Tyrode’s solution (Sigma-Aldrich, T1788). The embryos were then placed on agar in KSOM. Both blastomeres from each embryo were used for aggregation, generating double-sized chimeras. This approach was crucial, as blastomeres at the two-cell stage may vary substantially in their ability to form a blastocyst when separated, which could affect the results (*64*). Two types of dishes were tested: the Micro-Droplet culture dish (16003, VitroLife) and the GPS dish Embryo-Corral (EMBC-010, Life Global), with better aggregation efficiency to a single expanded blastocyst stage observed in the latter. A control and an aneuploid embryo were then aggregated together by gently bringing the blastomeres together using mouth pipetting at the tip of the pyramidal well. The Corral dishes contained 100 μl of preincubated KSOM medium per four-chamber well, covered with 100 μl of mineral oil (Origio, ART-4008-5P). The dishes were incubated at 37°C with 5% CO_2_ until 6 p.m. at E4.5. At this point, mature embryos were fixed in 4% PFA (freshly made from powder, Sigma-Aldrich, P6148) and incubated for 30 min at room temperature.

The embryos were then washed into PBT (PBS supplemented with 0.1% Triton X-100) and transferred to a well of a Nuncleon plate with 400 μl of PBT. The plate was sealed with Microseal “B” (MSB1001, Bio-Rad) and stored at 4°C until further use. On the day of immunofluorescence, the aggregates were then blocked in 3% (w/v) BSA-PBS for 30 min and incubated in Cdx2 antibody (1:400 dilution; AF3665-SP, R&D) overnight at 4°C. After washes the next day, the aggregates were incubated for 3 hours at room temperature with Alexa Fluor A647 anti-goat at 1:200 and DAPI. In the initial experiments, to assess whether the aggregates have correct lineage allocation (fig. S9C), also Gata4 (Invitrogen, 14-9980-80) and Sox2 (Abcam, ab97959) antibodies with corresponding secondaries were used to identify the other two lineages. Embryos were imaged using confocal microscopy and stored at 4°C until three-dimensional (3D)–FISH.

### 3D chromosome painting to determine the degree of mosaicism in aggregated blastocysts

On the day of 3D-FISH, the aggregated blastocysts were placed into a permeabilization solution [0.5% Triton X and polyvinyl pyrrolidone (PVP) (1 mg/ml) in PBS] on agar plates for 2 hours at 37°C based on a method adapted from the following protocol (*83*). Meanwhile, a 35-mm round dish was incubated at 37°C with three droplets under mineral oil: prehybridization mix {50% formamide: 50% hybridization buffer [20% dextran sulfate, 4× SSC, 1 mM EDTA, 0.1% Triton X-100, PVP (2 mg/ml), 1 mg/ml BSA]}, blocking mix [10 μg Cot-1 DNA (Invitrogen, 18440-016) and 5 μg of salmon sperm DNA (Invitrogen, 1563201)—premixed and precipitated in ethanol at 4°C, air dried, and resuspended in 5 μl of formamide], and 1 μl of each of the two DNA FISH probes (XMP Y Orange D-1421-050-OR and XMP X Green D-1420-050-FI, Metasystems), premixed. After the permeabilization step, the embryos were washed with PBS-Tp [0.05% Triton X and PVP (1 mg/ml) in PBS] and placed for at least 20 min in a drop of prehybridization mix. Next, the embryos were placed in the blocking mix (10 μg Cot-1 DNA and 5 μg of salmon sperm DNA, preprecipitated in ethanol at 4°C, air dried, and resuspended in 5 μl formamide) for at least 2 hours. After the Cot-1 incubation, the entire 35-mm dish was placed on a heat block for 10 min at 83°C, ensuring that the probes and embryos denature separately. Afterward, the dish was placed at 37°C for 2 to 5 hours. Then, the embryos were transferred from the blocking mix to the droplet containing the FISH probes and incubated at 37°C for one to two nights. After the hybridization, the following washes were done: a brief wash in 2× SSC-Tp at room temperature, followed by a 30-min wash in 0.2× SSC-Tp solution [each with 0.5% Triton X-100, PVP (1 mg/ml)] in a Nuncleon plate placed on a heat block at 45°C. Afterward, the embryos were incubated in PBT-Tp with DAPI to stain the nuclei and were placed in a droplet with VECTASHIELD Antifade Mounting Medium (2B Scientific, H-1000) in a glass-bottom dish (35-mm dish with 14-mm well, #1.5 glass; D35-14-1.5-N, Cellvis). The embryos were then covered with a 13-mm round glass coverslip (VWR) to ensure good depth of imaging. Embryos position was marked with a pen to allow an easy identification during imaging.

### Confocal fixed cell microscopy

Confocal images were acquired using a Zeiss LSM 880 confocal microscope. For chromosome spreads fixed on glass slides, a Plan-Apochromat 63×/1.4 oil immersion objective was used. In most experiments, 15 images were acquired with 0.63-μm optical sectioning. Care was taken to avoid fluorescent signal oversaturation. To prevent any potential differences in how chromosomes respond to the DNA FISH protocol, confocal imaging for quantification of immunofluorescence signal in all experiments apart from the initial trials was performed before DNA FISH and the second round of confocal imaging. For imaging intact spindles in zygotes and mosaic aggregates, the Zeiss LSM 880 confocal microscope with a 40× C-Apochromat 1.2–numerical aperture (NA) water-immersion objective was used (with optical sectioning of 0.17 and 2 μm, respectively). All images where the intensity of fluorescent signal was compared were obtained using the confocal mode. In cases where chromosome counting on intact spindles was performed, Airyscan mode was used for some imaging sessions.

### Live light-sheet imaging in microinjected zygotes

For light-sheet imaging, zygotes were imaged in 100 μl of medium under paraffin oil in a multiwell sample holder with four compartments (Viventis Microscopy Sàrl). Four small indentations were created in each compartment, typically coculturing around 10 zygotes per indentation. Imaging was performed using the LS1 Live system (Viventis Microscopy Sàrl), equipped with an environmental incubator box and a water-dipping objective. The light-sheet thickness was 2.2 μm. Images were acquired with a temporal resolution of 5 min, capturing 111 sections with a 1-μm thickness. Laser lines of 488 and 561 nm were used for excitation, and detection was achieved with a green fluorescent protein/mCherry (DBP) emission filter.

### Live light-sheet imaging to monitor cell cycle length and ploidy determination

Matings of wild-type and X^y^ males with wild-type females (all MF1 background) were set up in parallel. In cases where at least one wild type and one X^y^ male mated on the same day, females were euthanized in the early morning of 2nd day postfertilization. Embryos were retrieved in FHM medium, and subsequently exposed to pulse labeling with SPY555-DNA (Spirochrome, 1:1000 in KSOM) and SPY620-Actin (1:2000 in KSOM) for 1-hour in Nuncleon plates. Imaging was performed using the LS1 Live system (Viventis Microscopy Sàrl) in small customized indentations of the chamber. The imaging medium was KSOM, supplemented with SPY dyes as above at 1:5000. The light-sheet thickness was 2.2 μm. Images were acquired with a temporal resolution of 7 min, capturing 25 sections with a 5-μm thickness. After imaging, embryos were fixed and exposed to DNA FISH protocol to retrospectively distinguish between XY and XO embryos.

### Analysis of normalized CENP-A signal intensity

Confocal images used for quantification were acquired in 16-bit mode. CENP-A signal intensity was measured in Fiji using a sum projection of all slices encompassing all chromosomes. Channels were split, and the thresholding tool was applied to identify all centromeres. The watershed filter was used to separate overlapping binary objects. Afterward, signal intensity measurements of the identified objects were taken from a duplicated 16-bit image. The measurements, including minimum and maximum values, XY coordinates, Feret diameter, and raw integrated density, were exported to Excel. Overlapping signals were manually split when necessary. For CENP-A intensity, the average signal intensity across all centromeres in a given zygote was calculated, and individual centromere values were normalized to this average at the level of an individual zygote. Additional channels, such as H3K9me3, were used for further analysis. Using the object map generated in Fiji, each centromere was manually assigned as either maternal or paternal. Following DNA FISH and a second round of confocal imaging, Y and X chromosome centromeres were retrospectively identified.

### Analysis of difference in fluorescence intensity across sister centromeres

The difference in CENP-A intensity across sisters was calculated as previously described for human cells (*39*). In brief, the intensity per centromere was calculated as above on the basis of sum intensity projections. Then, the percentage difference was calculated as (centromere A – centromere B)/(centromere A + centromere B).

### Comparison of absolute fluorescence intensity of CENP-A (young versus old), Smc3, and Sgo2

For CENP-A analysis in zygotes from young versus old mothers, zygotes from six young and six old mice were processed on various days. Slides were stored at −80°C until further use. The slides were then labeled and imaged in parallel using identical settings to ensure consistency. To quantify centromere signal ratios, a sum intensity Z-projection was generated using Fiji/ImageJ software. Squares of a constant diameter were drawn around individual centromeres, and the total centromere fluorescence intensity was calculated using the corrected total cell fluorescence (CTCF) formula: CTCF = integrated density – (area of selected cell X mean fluorescence of background readings). For the background, five independent measurements per image were taken and the average background value was calculated.

### Quantification of cohesion fatigue of maternal versus paternal chromosomes in response to different durations of microtubule pulling

Zygotes retrieved from individual mice were kept in separate droplets and then split into two groups. One group was processed using the chromosome spreading protocol on the evening of retrieval (10 p.m.), while the other group was processed 12 hours later (10 a.m. the following morning). This allowed the paired comparisons to be done at the level of zygotes coming from the same mouse, for multiple mice. Immunofluorescence microscopy (CENP-A, H3K9me3, and DAPI) and DNA FISH (X and Y chromosomes) were performed as described above.

Sister centromere pairs were identified using 3D analysis of chromosome architecture (DAPI) in Imaris (Bitplane). The center of each centromere was detected with subpixel accuracy using the automated spot detection function, which identifies local maxima. The distances between sister centromeres were measured by extracting the xyz coordinates of the kinetochore centers, defined by the automated spot detection function in Imaris. These coordinates were then used to calculate the distances between the sister centromeres using Microsoft Excel.

### Quantification of sensitivity and specificity of Y chromosome recognition based on the absence of the MajSatTALE-StayGold signal

Zygotes microinjected with MajSatTALE-StayGold signal were subjected to chromosome spreading and fluorescence labeling for H3K9me3, CENP-A, and DAPI. Centromere pairs were classified as MajSat positive or MajSat negative on the basis of the presence or absence of MajSat signal in between the two sister centromeres, defined as per CENP-A signal. Of the 1800 chromosomes analyzed, one exhibited abnormal morphology, indicating an incidence of 1 of 1800 (0.06%) artefacts due to chromosome signal or structural abnormalities. Autosomes were distinguished from the X and Y chromosomes based on the DNA FISH signal, which was visualized during the second round of confocal microscopy. This helped to accurately assign the chromosomes for further analysis.

### Quantification of zygote live imaging data

Images acquired through light-sheet microscopy were analyzed in Fiji. Zygotic maturity (PN1-5) was determined on the basis of the size and relative location of the parental pronuclei, as described previously. The timing of nuclear envelope breakdown was annotated for each of the pronuclei separately, and the mean timing was then calculated. Anaphase was defined as the first time point at which chromosome separation became visible. Polar and lagging chromosomes were quantified using maximum intensity projections of all z-stacks recorded. Only spindles sufficiently parallel to the imaging plane, allowing for precise analysis, were considered. In Fig. 4C, the category of “polar chromosomes” refers to zygotes in which at least one polar chromosome was present at the time of anaphase onset.

### Cell counting and clonal origin in embryo aggregation experiments

For total cell counting, z-projections encompassing the entire blastocyst were opened in Fiji, and the “Cell Counter” plugin was used to annotate all cells, including those at metaphase and intact nuclei. Only embryos that reached the blastocoel stage were included in the analysis. TE and ICM were differentiated on the basis of the presence or absence of the Cdx2 signal.

Genotypes and clonal origin were determined on the basis of the second round of labeling (DNA FISH) and imaging. The counting of euploid versus sex chromosome aneuploid cells was performed again using the Cell Counter plugin. For the control group, only XY-XX embryos were included in the analysis, as the clonal origin of cells could not be determined in XY-XY embryos.

### Determining cell cycle length in XY versus XO embryos

To assess the behavior of individual cells, lineage trees were generated for each blastomere using ImageJ. Because cell cycle length varies across successive mitoses, care was taken to compare blastomeres at equivalent developmental stages. Accordingly, daughter cell identity and the developmental stage of the mother cell were annotated for each tracked division. As embryos were retrieved on day 2 after a positive plug check, the complete cell cycle (first time frame of nuclear envelope reformation to next mitosis) was captured for most cells for the fifth cleavage (16- to 32-cell stage). Cell cycle duration was defined as the interval from the last time point after division but before nuclear envelope reformation to the first time point of nuclear envelope breakdown in the subsequent division. The genetic status of each embryo (XY or XO) was determined retrospectively following DNA FISH analysis.

### Statistical data analyses

All graphs were generated using Prism (GraphPad) software. Graphs show mean (line) and SD and are overlaid with individual data points. Statistical significance evaluations were all performed using the Prism software, with the exception of the Fisher Exact Test (SocStatistics). Each experiment was conducted at least in three experimental or biological replicates (see figure legends for details), with the exception of Fig. 5J, where embryos from four mice were imaged in two independent repetitions. Nonsignificant was defined as *P* > 0.05 and was indicated in the graphs as “ns.”

## References

[1] L. A. Forsberg, D. Gisselsson, J. P. Dumanski, Mosaicism in health and disease - Clones picking up speed. Nat. Rev. Genet. 18, 128–142 (2017). 10.1038/nrg.2016.145 27941868

[2] X. Guo, X. Dai, T. Zhou, H. Wang, J. Ni, J. Xue, X. Wang, Mosaic loss of human Y chromosome: What, how and why. Hum. Genet. 139, 421–446 (2020). 10.1007/s00439-020-02114-w 32020362

[3] D. Fachinetti, J. S. Han, M. A. McMahon, P. Ly, A. Abdullah, A. J. Wong, D. W. Cleveland, DNA sequence-specific binding of CENP-B enhances the fidelity of human centromere function. Dev. Cell 33, 314–327 (2015). 10.1016/j.devcel.2015.03.020 PMC4421092 25942623

[4] S. Hoffmann, H. M. Izquierdo, R. Gamba, F. Chardon, M. Dumont, V. Keizer, S. Herve, S. M. McNulty, B. A. Sullivan, N. Manel, D. Fachinetti, A genetic memory initiates the epigenetic loop necessary to preserve centromere position. EMBO J. 39, e105505 (2020). 10.15252/embj.2020105505 PMC7560200 32945564

[5] S. J. Klaasen, G. Kops, Chromosome inequality: Causes and consequences of non-random segregation errors in mitosis and meiosis. Cells 11, 3564 (2022). 10.3390/cells11223564 PMC9688425 36428993

[6] P. Ly, L. S. Teitz, D. H. Kim, O. Shoshani, H. Skaletsky, D. Fachinetti, D. C. Page, D. W. Cleveland, Selective Y centromere inactivation triggers chromosome shattering in micronuclei and repair by non-homologous end joining. Nat. Cell Biol. 19, 68–75 (2017). 10.1038/ncb3450 PMC5539760 27918550

[7] D. Broccoli, O. J. Miller, D. A. Miller, Relationship of mouse minor satellite DNA to centromere activity. Cytogenet. Cell Genet. 54, 182–186 (1990). 10.1159/000132989 2265565

[8] R. Gamba, D. Fachinetti, From evolution to function: Two sides of the same CENP-B coin? Exp. Cell Res. 390, 111959 (2020). 10.1016/j.yexcr.2020.111959 32173469

[9] R. C. Allshire, G. H. Karpen, Epigenetic regulation of centromeric chromatin: Old dogs, new tricks? Nat. Rev. Genet. 9, 923–937 (2008). 10.1038/nrg2466 PMC2586333 19002142

[10] W. C. Earnshaw, N. Rothfield, Identification of a family of human centromere proteins using autoimmune sera from patients with scleroderma. Chromosoma 91, 313–321 (1985). 10.1007/BF00328227 2579778

[11] K. L. McKinley, I. M. Cheeseman, The molecular basis for centromere identity and function. Nat. Rev. Mol. Cell Biol. 17, 16–29 (2016). 10.1038/nrm.2015.5 PMC8603311 26601620

[12] B. G. Mellone, D. Fachinetti, Diverse mechanisms of centromere specification. Curr. Biol. 31, R1491–R1504 (2021). 10.1016/j.cub.2021.09.083 PMC8820161 34813757

[13] D. Drpic, A. C. Almeida, P. Aguiar, F. Renda, J. Damas, H. A. Lewin, D. M. Larkin, A. Khodjakov, H. Maiato, Chromosome segregation is biased by kinetochore size. Curr. Biol. 28, 1344–1356.e5 (2018). 10.1016/j.cub.2018.03.023 PMC5954971 29706521

[14] M. Dumont, R. Gamba, P. Gestraud, S. Klaasen, J. T. Worrall, S. G. De Vries, V. Boudreau, C. Salinas-Luypaert, P. S. Maddox, S. M. Lens, G. J. Kops, S. E. McClelland, K. H. Miga, D. Fachinetti, Human chromosome-specific aneuploidy is influenced by DNA-dependent centromeric features. EMBO J. 39, e102924 (2020). 10.15252/embj.2019102924 PMC6960447 31750958

[15] A. D. McAinsh, A. L. Marston, The four causes: The functional architecture of centromeres and kinetochores. Annu. Rev. Genet. 56, 279–314 (2022). 10.1146/annurev-genet-072820-034559 PMC7614391 36055650

[16] K. Eggan, A. Rode, I. Jentsch, C. Samuel, T. Hennek, H. Tintrup, B. Zevnik, J. Erwin, J. Loring, L. Jackson-Grusby, M. R. Speicher, R. Kuehn, R. Jaenisch, Male and female mice derived from the same embryonic stem cell clone by tetraploid embryo complementation. Nat. Biotechnol. 20, 455–459 (2002). 10.1038/nbt0502-455 11981557

[17] K. Murakami, N. Hamazaki, N. Hamada, G. Nagamatsu, I. Okamoto, H. Ohta, Y. Nosaka, Y. Ishikura, T. S. Kitajima, Y. Semba, Y. Kunisaki, F. Arai, K. Akashi, M. Saitou, K. Kato, K. Hayashi, Generation of functional oocytes from male mice in vitro. Nature 615, 900–906 (2023). 10.1038/s41586-023-05834-x 36922585

[18] H. Hall, P. Hunt, T. Hassold, Meiosis and sex chromosome aneuploidy: How meiotic errors cause aneuploidy; how aneuploidy causes meiotic errors. Curr. Opin. Genet. Dev. 16, 323–329 (2006). 10.1016/j.gde.2006.04.011 16647844

[19] J. F. Hughes, D. C. Page, The biology and evolution of mammalian Y chromosomes. Annu. Rev. Genet. 49, 507–527 (2015). 10.1146/annurev-genet-112414-055311 26442847

[20] R. Migale, M. Neumann, R. Lovell-Badge, Long-range regulation of key sex determination genes. Sex. Dev. 15, 360–380 (2022). 10.1159/000519891 34753143

[21] J. Subrini, J. Turner, Y chromosome functions in mammalian spermatogenesis. eLife 10, e67345 (2021). 10.7554/eLife.67345 PMC8489898 34606444

[22] S. I. Nagaoka, T. J. Hassold, P. A. Hunt, Human aneuploidy: Mechanisms and new insights into an age-old problem. Nat. Rev. Genet. 13, 493–504 (2012). 10.1038/nrg3245 PMC3551553 22705668

[23] M. Herbert, D. Kalleas, D. Cooney, M. Lamb, L. Lister, Meiosis and maternal aging: Insights from aneuploid oocytes and trisomy births. Cold Spring Harb. Perspect. Biol. 7, a017970 (2015). 10.1101/cshperspect.a017970 PMC4382745 25833844

[24] J. R. Gruhn, A. P. Zielinska, V. Shukla, R. Blanshard, A. Capalbo, D. Cimadomo, D. Nikiforov, A. C. Chan, L. J. Newnham, I. Vogel, C. Scarica, M. Krapchev, D. Taylor, S. G. Kristensen, J. Cheng, E. Ernst, A. B. Bjorn, L. B. Colmorn, M. Blayney, K. Elder, J. Liss, G. Hartshorne, M. L. Grondahl, L. Rienzi, F. Ubaldi, R. McCoy, K. Lukaszuk, C. Y. Andersen, M. Schuh, E. R. Hoffmann, Chromosome errors in human eggs shape natural fertility over reproductive life span. Science 365, 1466–1469 (2019). 10.1126/science.aav7321 PMC7212007 31604276

[25] A. Uematsu, T. Yorifuji, J. Muroi, M. Kawai, M. Mamada, M. Kaji, C. Yamanaka, T. Momoi, T. Nakahata, Parental origin of normal X chromosomes in Turner syndrome patients with various karyotypes: Implications for the mechanism leading to generation of a 45, X karyotype. Am. J. Med. Genet. 111, 134–139 (2002). 10.1002/ajmg.10506 12210339

[26] T. Hassold, D. Pettay, A. Robinson, I. Uchida, Molecular studies of parental origin and mosaicism in 45, X conceptuses. Hum. Genet. 89, 647–652 (1992). 10.1007/BF00221956 1511983

[27] R. De La Fuente, C. Baumann, M. M. Viveiros, ATRX contributes to epigenetic asymmetry and silencing of major satellite transcripts in the maternal genome of the mouse embryo. Development 142, 1806–1817 (2015). 10.1242/dev.118927 PMC4440925 25926359

[28] A. Das, A. Iwata-Otsubo, A. Destouni, J. M. Dawicki-McKenna, K. G. Boese, B. E. Black, M. A. Lampson, Epigenetic, genetic and maternal effects enable stable centromere inheritance. Nat. Cell Biol. 24, 748–756 (2022). 10.1038/s41556-022-00897-w PMC9107508 35534577

[29] A. Das, B. E. Black, M. A. Lampson, Maternal inheritance of centromeres through the germline. Curr. Top. Dev. Biol. 140, 35–54 (2020). 10.1016/bs.ctdb.2020.03.004 32591081

[30] C. A. Tower, G. Manske, E. L. Ferrell, D. N. Anbarci, K. Jorgensen, B. Ma, M. Aboelenain, R. Ranjan, S. Chakraborty, L. Moritz, A. Das, M. Boiani, B. E. Black, S. Chavez, E. E. Marsh, A. Shikanov, K. Schindler, X. Chen, S. S. Hammoud, Maternal CENP-C restores centromere symmetry in mammalian zygotes to ensure proper chromosome segregation. Dev. Cell 61, 146–163.e10 (2026). 10.1016/j.devcel.2025.08.017 PMC12479092 40997799

[31] S. Santaguida, A. Musacchio, The life and miracles of kinetochores. EMBO J. 28, 2511–2531 (2009). 10.1038/emboj.2009.173 PMC2722247 19629042

[32] A. Burton, V. Brochard, C. Galan, E. R. Ruiz-Morales, Q. Rovira, D. Rodriguez-Terrones, K. Kruse, S. Le Gras, V. S. Udayakumar, H. G. Chin, A. Eid, X. Liu, C. Wang, S. Gao, S. Pradhan, J. M. Vaquerizas, N. Beaujean, T. Jenuwein, M. E. Torres-Padilla, Heterochromatin establishment during early mammalian development is regulated by pericentromeric RNA and characterized by non-repressive H3K9me3. Nat. Cell Biol. 22, 767–778 (2020). 10.1038/s41556-020-0536-6 PMC7610380 32601371

[33] H. Liu, J. M. Kim, F. Aoki, Regulation of histone H3 lysine 9 methylation in oocytes and early pre-implantation embryos. Development 131, 2269–2280 (2004). 10.1242/dev.01116 15102709

[34] A. Allais, G. FitzHarris, Absence of a robust mitotic timer mechanism in early preimplantation mouse embryos leads to chromosome instability. Development 149, dev200391 (2022). 10.1242/dev.200391 35771634

[35] K. L. Sackton, N. Dimova, X. Zeng, W. Tian, M. Zhang, T. B. Sackton, J. Meaders, K. L. Pfaff, F. Sigoillot, H. Yu, X. Luo, R. W. King, Synergistic blockade of mitotic exit by two chemical inhibitors of the APC/C. Nature 514, 646–649 (2014). 10.1038/nature13660 PMC4214887 25156254

[36] D. L. Bodor, J. F. Mata, M. Sergeev, A. F. David, K. J. Salimian, T. Panchenko, D. W. Cleveland, B. E. Black, J. V. Shah, L. E. Jansen, The quantitative architecture of centromeric chromatin. eLife 3, e02137 (2014). 10.7554/eLife.02137 PMC4091408 25027692

[37] D. V. Irvine, D. J. Amor, J. Perry, N. Sirvent, F. Pedeutour, K. H. Choo, R. Saffery, Chromosome size and origin as determinants of the level of CENP-A incorporation into human centromeres. Chromosome Res. 12, 805–815 (2004). 10.1007/s10577-005-5377-4 15702419

[38] Y. Watanabe, Shugoshin: Guardian spirit at the centromere. Curr. Opin. Cell Biol. 17, 590–595 (2005). 10.1016/j.ceb.2005.10.003 16229998

[39] J. E. Ross, K. S. Woodlief, B. A. Sullivan, Inheritance of the CENP-A chromatin domain is spatially and temporally constrained at human centromeres. Epigenetics Chromatin 9, 20 (2016). 10.1186/s13072-016-0071-7 PMC4888493 27252782

[40] J. Gubbay, J. Collignon, P. Koopman, B. Capel, A. Economou, A. Munsterberg, N. Vivian, P. Goodfellow, R. Lovell-Badge, A gene mapping to the sex-determining region of the mouse Y chromosome is a member of a novel family of embryonically expressed genes. Nature 346, 245–250 (1990). 10.1038/346245a0 2374589

[41] Y. Itoh, R. Mackie, K. Kampf, S. Domadia, J. D. Brown, R. O’Neill, A. P. Arnold, Four core genotypes mouse model: Localization of the Sry transgene and bioassay for testicular hormone levels. BMC. Res. Notes 8, 69 (2015). 10.1186/s13104-015-0986-2 PMC4354741 25870930

[42] A. Das, K. G. Boese, K. Tachibana, S. H. Baek, M. A. Lampson, B. E. Black, Centromere-specifying nucleosomes persist in aging mouse oocytes in the absence of nascent assembly. Curr. Biol. 33, 3759–3765.e3 (2023). 10.1016/j.cub.2023.07.032 PMC10528140 37582374

[43] D. Clift, M. Schuh, Restarting life: Fertilization and the transition from meiosis to mitosis. Nat. Rev. Mol. Cell Biol. 14, 549–562 (2013). 10.1038/nrm3643 PMC4021448 23942453

[44] E. M. Smoak, P. Stein, R. M. Schultz, M. A. Lampson, B. E. Black, Long-term retention of CENP-a nucleosomes in mammalian oocytes underpins transgenerational inheritance of centromere identity. Curr. Biol. 26, 1110–1116 (2016). 10.1016/j.cub.2016.02.061 PMC4846481 27040782

[45] D. L. Bodor, L. P. Valente, J. F. Mata, B. E. Black, L. E. Jansen, Assembly in G1 phase and long-term stability are unique intrinsic features of CENP-A nucleosomes. Mol. Biol. Cell 24, 923–932 (2013). 10.1091/mbc.E13-01-0034 PMC3608502 23363600

[46] L. E. Jansen, B. E. Black, D. R. Foltz, D. W. Cleveland, Propagation of centromeric chromatin requires exit from mitosis. J. Cell Biol. 176, 795–805 (2007). 10.1083/jcb.200701066 PMC2064054 17339380

[47] L. Yin, A. I. Mihajlovic, G. Yang, G. FitzHarris, Kinetochore deterioration concommitant with centromere weakening during aging in mouse oocyte meiosis-I. FASEB J. 37, e22922 (2023). 10.1096/fj.202300062R 37078553

[48] C. J. Bean, P. A. Hunt, E. A. Millie, T. J. Hassold, Analysis of a malsegregating mouse Y chromosome: Evidence that the earliest cleavage divisions of the mammalian embryo are non-disjunction-prone. Hum. Mol. Genet. 10, 963–972 (2001). 10.1093/hmg/10.9.963 11309370

[49] A. N. Graham, P. Kalitsis, Chromosome Y centromere array deletion leads to impaired centromere function. PLOS ONE 9, e86875 (2014). 10.1371/journal.pone.0086875 PMC3899357 24466276

[50] J. Reichmann, B. Nijmeijer, M. J. Hossain, M. Eguren, I. Schneider, A. Z. Politi, M. J. Roberti, L. Hufnagel, T. Hiiragi, J. Ellenberg, Dual-spindle formation in zygotes keeps parental genomes apart in early mammalian embryos. Science 361, 189–193 (2018). 10.1126/science.aar7462 30002254

[51] M. Guenatri, D. Bailly, C. Maison, G. Almouzni, Mouse centric and pericentric satellite repeats form distinct functional heterochromatin. J. Cell Biol. 166, 493–505 (2004). 10.1083/jcb.200403109 PMC2172221 15302854

[52] Y. Miyanari, C. Ziegler-Birling, M. E. Torres-Padilla, Live visualization of chromatin dynamics with fluorescent TALEs. Nat. Struct. Mol. Biol. 20, 1321–1324 (2013). 10.1038/nsmb.2680 24096363

[53] J. R. Daum, T. A. Potapova, S. Sivakumar, J. J. Daniel, J. N. Flynn, S. Rankin, G. J. Gorbsky, Cohesion fatigue induces chromatid separation in cells delayed at metaphase. Curr. Biol. 21, 1018–1024 (2011). 10.1016/j.cub.2011.05.032 PMC3119564 21658943

[54] H. Sapkota, E. Wasiak, J. R. Daum, G. J. Gorbsky, Multiple determinants and consequences of cohesion fatigue in mammalian cells. Mol. Biol. Cell 29, 1811–1824 (2018). 10.1091/mbc.E18-05-0315 PMC6085821 29846129

[55] J. T. Worrall, N. Tamura, A. Mazzagatti, N. Shaikh, T. van Lingen, B. Bakker, D. C. J. Spierings, E. Vladimirou, F. Foijer, S. E. McClelland, Non-random mis-segregation of human chromosomes. Cell Rep. 23, 3366–3380 (2018). 10.1016/j.celrep.2018.05.047 PMC6019738 29898405

[56] Y. Yun, S. I. Lane, K. T. Jones, Premature dyad separation in meiosis II is the major segregation error with maternal age in mouse oocytes. Development 141, 199–208 (2014). 10.1242/dev.100206 PMC3913075 24346700

[57] C. Vazquez-Diez, L. M. G. Paim, G. FitzHarris, Cell-size-independent spindle checkpoint failure underlies chromosome segregation error in mouse embryos. Curr. Biol. 29, 865–873.e3 (2019). 10.1016/j.cub.2018.12.042 30773364

[58] K. Vukusic, I. M. Tolic, Polar chromosomes-challenges of a risky path. Cells 11, 1531 (2022). 10.3390/cells11091531 PMC9101661 35563837

[59] H. Bolton, S. J. L. Graham, N. Van der Aa, P. Kumar, K. Theunis, E. Fernandez Gallardo, T. Voet, M. Zernicka-Goetz, Mouse model of chromosome mosaicism reveals lineage-specific depletion of aneuploid cells and normal developmental potential. Nat. Commun. 7, 11165 (2016). 10.1038/ncomms11165 PMC4820631 27021558

[60] S. Singla, L. K. Iwamoto-Stohl, M. Zhu, M. Zernicka-Goetz, Autophagy-mediated apoptosis eliminates aneuploid cells in a mouse model of chromosome mosaicism. Nat. Commun. 11, 2958 (2020). 10.1038/s41467-020-16796-3 PMC7290028 32528010

[61] M. Yang, T. Rito, J. Metzger, J. Naftaly, R. Soman, J. Hu, D. F. Albertini, D. H. Barad, A. H. Brivanlou, N. Gleicher, Depletion of aneuploid cells in human embryos and gastruloids. Nat. Cell Biol. 23, 314–321 (2021). 10.1038/s41556-021-00660-7 33837289

[62] P. S. Burgoyne, S. K. Mahadevaiah, J. Perry, S. J. Palmer, A. Ashworth, The Y* rearrangement in mice: New insights into a perplexing PAR. Cytogenet. Cell Genet. 80, 37–40 (1998). 10.1159/000014954 9678332

[63] E. Casser, S. Israel, A. Witten, K. Schulte, S. Schlatt, V. Nordhoff, M. Boiani, Totipotency segregates between the sister blastomeres of two-cell stage mouse embryos. Sci. Rep. 7, 8299 (2017). 10.1038/s41598-017-08266-6 PMC5557898 28811525

[64] S. Junyent, M. Meglicki, R. Vetter, R. Mandelbaum, C. King, E. M. Patel, L. Iwamoto-Stohl, C. Reynell, D. Y. Chen, P. Rubino, N. Arrach, R. J. Paulson, D. Iber, M. Zernicka-Goetz, The first two blastomeres contribute unequally to the human embryo. Cell 187, 2838–2854.e17 (2024). 10.1016/j.cell.2024.04.029 38744282

[65] A. Musacchio, E. D. Salmon, The spindle-assembly checkpoint in space and time. Nat. Rev. Mol. Cell Biol. 8, 379–393 (2007). 10.1038/nrm2163 17426725

[66] J. Rueness, L. Vatten, A. Eskild, The human sex ratio: Effects of maternal age. Hum. Reprod. 27, 283–287 (2012). 10.1093/humrep/der347 22025225

[67] E. Bellou, A. P. Zielinska, E. U. Monnich, N. Schweizer, A. Z. Politi, A. Wellecke, C. Sibold, A. Tandler-Schneider, M. Schuh, Chromosome architecture and low cohesion bias acrocentric chromosomes towards aneuploidy during mammalian meiosis. Nat. Commun. 15, 10713 (2024). 10.1038/s41467-024-54659-3 PMC11666783 39715766

[68] J. L. Simpson, M. M. Lebeau, Gonadal and statural determinants on the X chromosome and their relationship to in vitro studies showing prolonged cell cycles in 45,X; 46,X,del(X)(p11); 46,X,del(X)(q13); and 46,X,del(X)(q22) fibroblasts. Am. J. Obstet. Gynecol. 141, 930–940 (1981). 10.1016/s0002-9378(16)32685-0 7315923

[69] M. S. Verp, B. Rosinsky, M. M. Le Beau, A. O. Martin, R. Kaplan, C. B. Wallemark, L. Otano, J. L. Simpson, Growth disadvantage of 45,X and 46,X,del (X) (p11) fibroblasts. Clin. Genet. 33, 277–285 (1988). 10.1111/j.1399-0004.1988.tb03449.x 3359684

[70] A. M. Denes, K. Landin-Wilhelmsen, Y. Wettergren, I. Bryman, C. Hanson, The proportion of diploid 46,xx cells increases with time in women with turner syndrome—A 10-year follow-up study. Genet. Test. Mol. Biomarkers 19, 82–87 (2015). 10.1089/gtmb.2014.0240 PMC4313393 25587646

[71] D. Serapinas, D. Bartkeviciene, E. Valantinaviciene, E. Machtejeviene, Normal newborn with prenatal suspicion of X chromosome monosomy due to confined placental mosaicism. Arch. Argent. Pediatr. 114, e362–e365 (2016). 10.5546/aap.2016.eng.e362 27606664

[72] E. Flori, B. Doray, E. Gautier, M. Kohler, P. Ernault, J. Flori, J. M. Costa, Circulating cell-free fetal DNA in maternal serum appears to originate from cyto- and syncytio-trophoblastic cells. Case report. Hum. Reprod. 19, 723–724 (2004). 10.1093/humrep/deh117 14998976

[73] C. E. Currie, E. Ford, L. Benham Whyte, D. M. Taylor, B. P. Mihalas, M. Erent, A. L. Marston, G. M. Hartshorne, A. D. McAinsh, The first mitotic division of human embryos is highly error prone. Nat. Commun. 13, 6755 (2022). 10.1038/s41467-022-34294-6 PMC9643329 36347869

[74] R. C. McCoy, L. J. Newnham, C. S. Ottolini, E. R. Hoffmann, K. Chatzimeletiou, O. E. Cornejo, Q. Zhan, N. Zaninovic, Z. Rosenwaks, D. A. Petrov, Z. P. Demko, S. Sigurjonsson, A. H. Handyside, Tripolar chromosome segregation drives the association between maternal genotype at variants spanning PLK4 and aneuploidy in human preimplantation embryos. Hum. Mol. Genet. 27, 2573–2585 (2018). 10.1093/hmg/ddy147 PMC6030883 29688390

[75] L. Wartosch, K. Schindler, M. Schuh, J. R. Gruhn, E. R. Hoffmann, R. C. McCoy, J. Xing, Origins and mechanisms leading to aneuploidy in human eggs. Prenat. Diagn. 41, 620–630 (2021). 10.1002/pd.5927 PMC8237340 33860956

[76] K. L. Palmerola, S. Amrane, A. De Los Angeles, S. Xu, N. Wang, J. de Pinho, M. V. Zuccaro, A. Taglialatela, D. J. Massey, J. Turocy, A. Robles, A. Subbiah, B. Prosser, R. Lobo, A. Ciccia, A. Koren, T. Baslan, D. Egli, Replication stress impairs chromosome segregation and preimplantation development in human embryos. Cell 185, 2988–3007.e20 (2022). 10.1016/j.cell.2022.06.028 35858625

[77] M. C. C. Silva, G. Wutz, K. Tachibana, J. M. Peters, Analysis of chromosomes from mouse oocytes and mammalian cultured cells by light microscopy. Methods Cell Biol. 144, 287–305 (2018). 10.1016/bs.mcb.2018.03.015 29804673

[78] J. Subrini, W. Varsally, I. B. Balsells, M. Bensberg, G. Sioutas, O. Ojarikre, V. Maciulyte, B. Gylemo, K. Crawley, K. Courtis, D. G. de Rooij, J. M. A. Turner, Systematic identification of Y-chromosome gene functions in mouse spermatogenesis. Science 387, 393–400 (2025). 10.1126/science.ads6495 PMC7617377 39847625

[79] H. Liu, Q. Qu, R. Warrington, A. Rice, N. Cheng, H. Yu, Mitotic transcription installs Sgo1 at centromeres to coordinate chromosome segregation. Mol. Cell 59, 426–436 (2015). 10.1016/j.molcel.2015.06.018 26190260

[80] K. Hayashi, M. Saitou, Generation of eggs from mouse embryonic stem cells and induced pluripotent stem cells. Nat. Protoc. 8, 1513–1524 (2013). 10.1038/nprot.2013.090 23845963

[81] T. Cermak, C. G. Starker, D. F. Voytas, Efficient design and assembly of custom TALENs using the Golden Gate platform. Methods Mol. Biol. 1239, 133–159 (2015). 10.1007/978-1-4939-1862-1_7 25408404

[82] A. Domingo-Muelas, R. M. Skory, A. A. Moverley, G. Ardestani, O. Pomp, C. Rubio, P. Tetlak, B. Hernandez, E. A. Rhon-Calderon, L. Navarro-Sanchez, C. M. Garcia-Pascual, S. Bissiere, M. S. Bartolomei, D. Sakkas, C. Simon, N. Plachta, Human embryo live imaging reveals nuclear DNA shedding during blastocyst expansion and biopsy. Cell 186, 3166–3181.e18 (2023). 10.1016/j.cell.2023.06.003 PMC11170958 37413989

[83] K. Ancelin, Y. Miyanari, O. Leroy, M. E. Torres-Padilla, E. Heard, Mapping of chromosome territories by 3D-chromosome painting during early mouse development. Methods Mol. Biol. 2214, 175–187 (2021). 10.1007/978-1-0716-0958-3_12 32944910

