# Supplementary material for: Y chromosome instability in the early mouse embryo
Source: Sci Adv. 2026 Nov 6;12(45):eaej3098. doi: 10.1126/sciadv.aej3098 (PMC13742188; doi:10.1126/sciadv.aej3098)
Supplement: Supplementary file 1 [file sciadv.aej3098_sm.pdf]

Science Advances
AAAS

Supplementary Materials for

Y chromosome instability in the early mouse embryo

Agata P. Zielinska *et al.*

Corresponding author: James M. A. Turner, james.turner@crick.ac.uk

Sci. Adv. **12**, eaej3098 (2026)
DOI: 10.1126/sciadv.aej3098

The PDF file includes:

Figs. S1 to S9
Legends for movies S1 to S6

Other Supplementary Material for this manuscript includes the following:

Movies S1 to S6

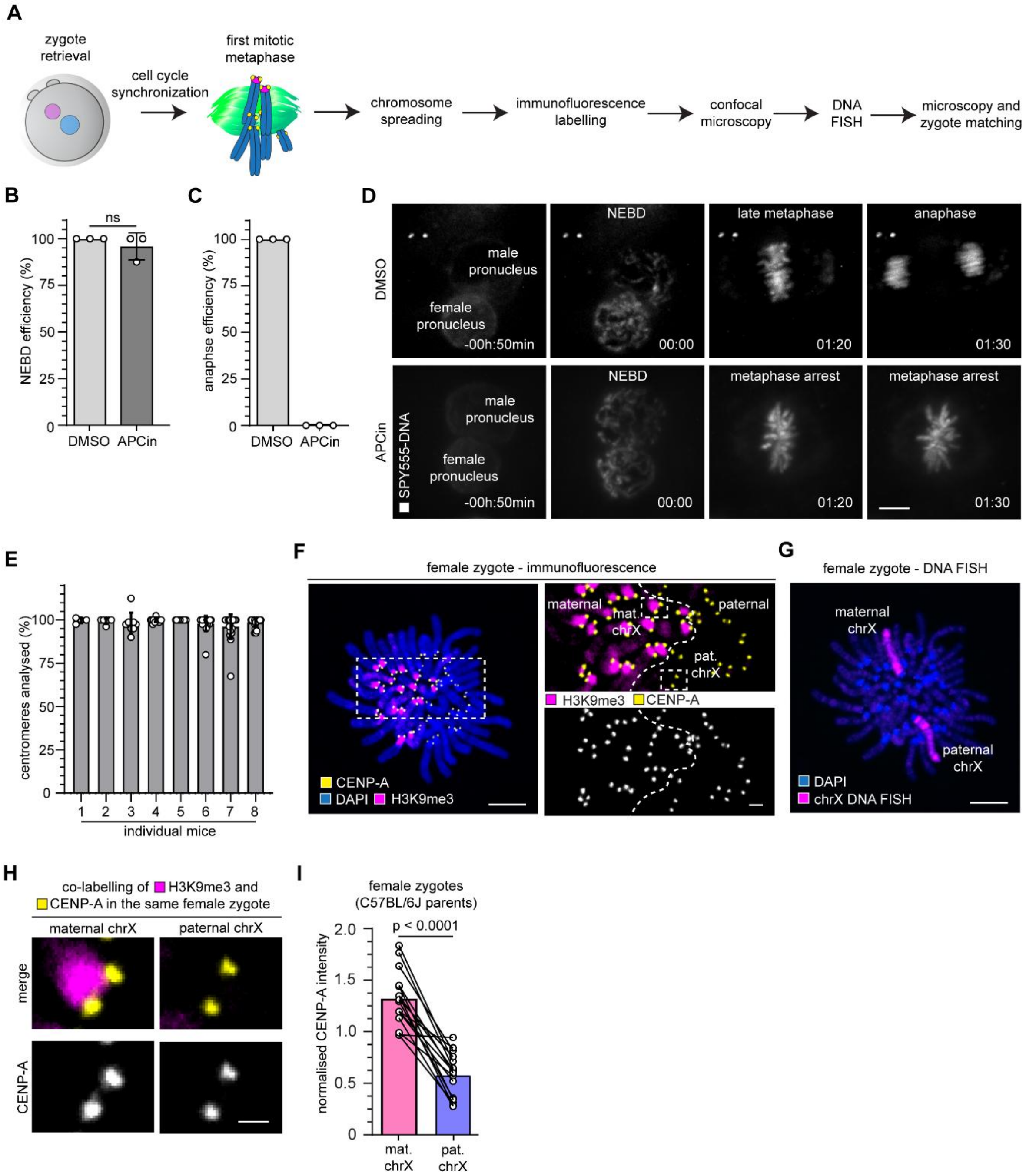

Fig. S1: Method for chromosome spreading in zygotes that enables analysis of specific, pre-defined individual chromosomes and reliable detection of >98% centromeres. (**A**) Scheme illustrating the steps of the co-immunofluorescence and DNA FISH protocol in mouse zygotes. (**B**) Quantification of fraction of live zygotes labelled as in (D) that progress through nuclear envelope breakdown (NEBD). Each dot represents one mouse. n = 45 zygotes from three mice imaged individually over two experimental repetitions. Statistical significance was evaluated using paired t-test. (**C**) Quantification of fraction of the live zygotes described in (B) that progress through anaphase. (**D**) Maximum intensity projected images of live zygotes cultured in the presence of DMSO (control) or APCin. Images were acquired every 10 minutes. Scalebar: 10 µm. (**E**) Quantification of the fraction of centromeres (out of total 80) which were available for analysis after chromosome spreading protocol as in (A). Each dot represents a single zygote. n = 121 zygotes from 8 mice. **(F)** Maximum intensity projection of chromosomes from the first embryonic metaphase in a female

mouse zygote from CBAxB6 F1 hybrid parents. H3K9me3 labelling was used to differentiate parental origin of chromosomes (H3K9me3+: maternal, H3K9me3-: paternal). Scalebars: large images 10 µm, insets 2 µm. (**G**) DNA FISH labelling of sex chromosomes in the same female zygote as in (F). Scalebar: 10 µm. (**H**) Insets show sex chromosome centromeres from panel (F). Scalebar: 1 µm. (**I**) Difference in centromere strength across maternal versus paternal X chromosome in the same zygote (C57BL/6J strain). n = 30 maternal ChrX and 32 paternal ChrX chromosomes from 7 experiments. Statistical significance was evaluated using paired t-test. Error bars show mean +/- SD.

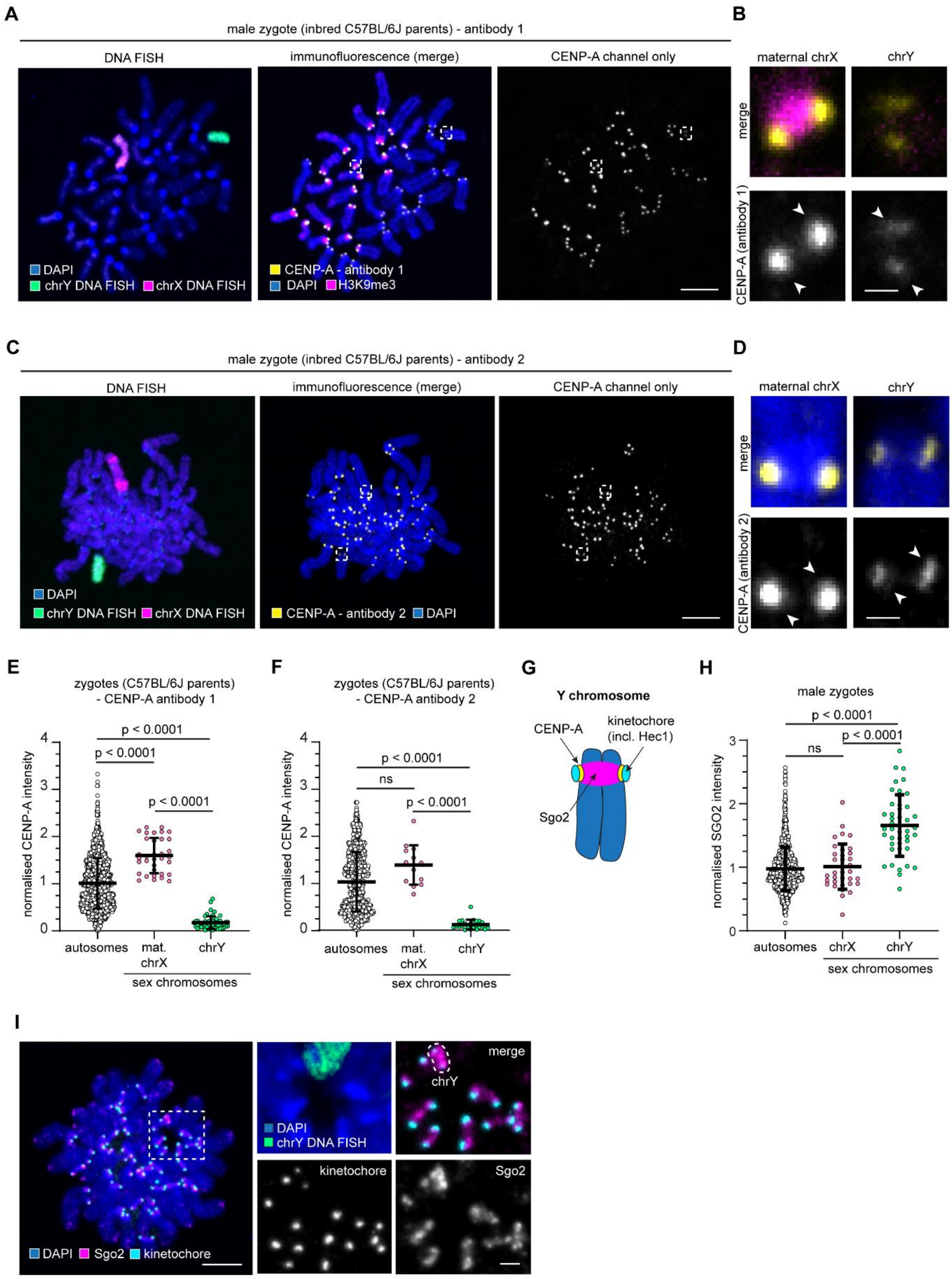

Fig. S2: Y chromosome centromeric weakness in zygotes is consistent across mouse strains and is more pronounced in C57BL/6J inbred mice. (**A**) Maximum intensity projection of chromosome spreads from the first embryonic metaphase of a naturally mated inbred C57BL/6J mouse. Panel on the left shows DNA FISH for sex chromosomes, while panel in the middle is an image of the same

zygote with CENP-A (antibody 1) and H3K9me3 antibody labelling. (**B**) Insets show a magnification of sex chromosome centromeres from panel (A). (**C**) Male zygote processed as in panel A, but using a different CENP-A antibody (antibody 2). (**D**) Insets show a magnification of sex chromosome centromeres from panel (C). (**E**) Quantification of normalised CENP-A sum fluorescence intensity of individual centromeres from naturally mated inbred mice of the C57BL/6J strain, where CENP-A is detected with antibody 1. Centromeres analysed: N = 6 experimental repetitions, n = 1,042 autosomes; 34 chrX centromeres; 52 chrY centromeres. (**F**) Quantification of normalised CENP-A sum fluorescence intensity in male zygotes as in (E), but using an alternative CENP-A antibody (antibody 2). Centromeres analysed: N = 3 experimental repetitions; 12 male zygotes, n = 902 autosomes; 14 chrX centromeres; 23 chrY centromeres. (**G**) Diagram illustrating the spatial distribution of selected centromeric factors (CENP-A and Sgo2) alongside kinetochore proteins. (**H**) Quantification of normalised Sgo2 sum fluorescence intensity across individual centromeres in zygotes (as shown in (G)). Centromeres analysed: n = 1,267 autosomes; 35 chrX; 45 chrY. N = 27 zygotes from 3 mice processed individually. Each dot represents a single centromere. (**I**) Maximum intensity projection of a chromosome spread from the first embryonic metaphase showing Sgo2 staining pattern. Boxes highlight regions that are magnified in the insets. Scale bars: 10 µm for large images, 1 µm for insets. Statistical significance was evaluated using Kruskal-Wallis test. Error bars show mean +/-SD.

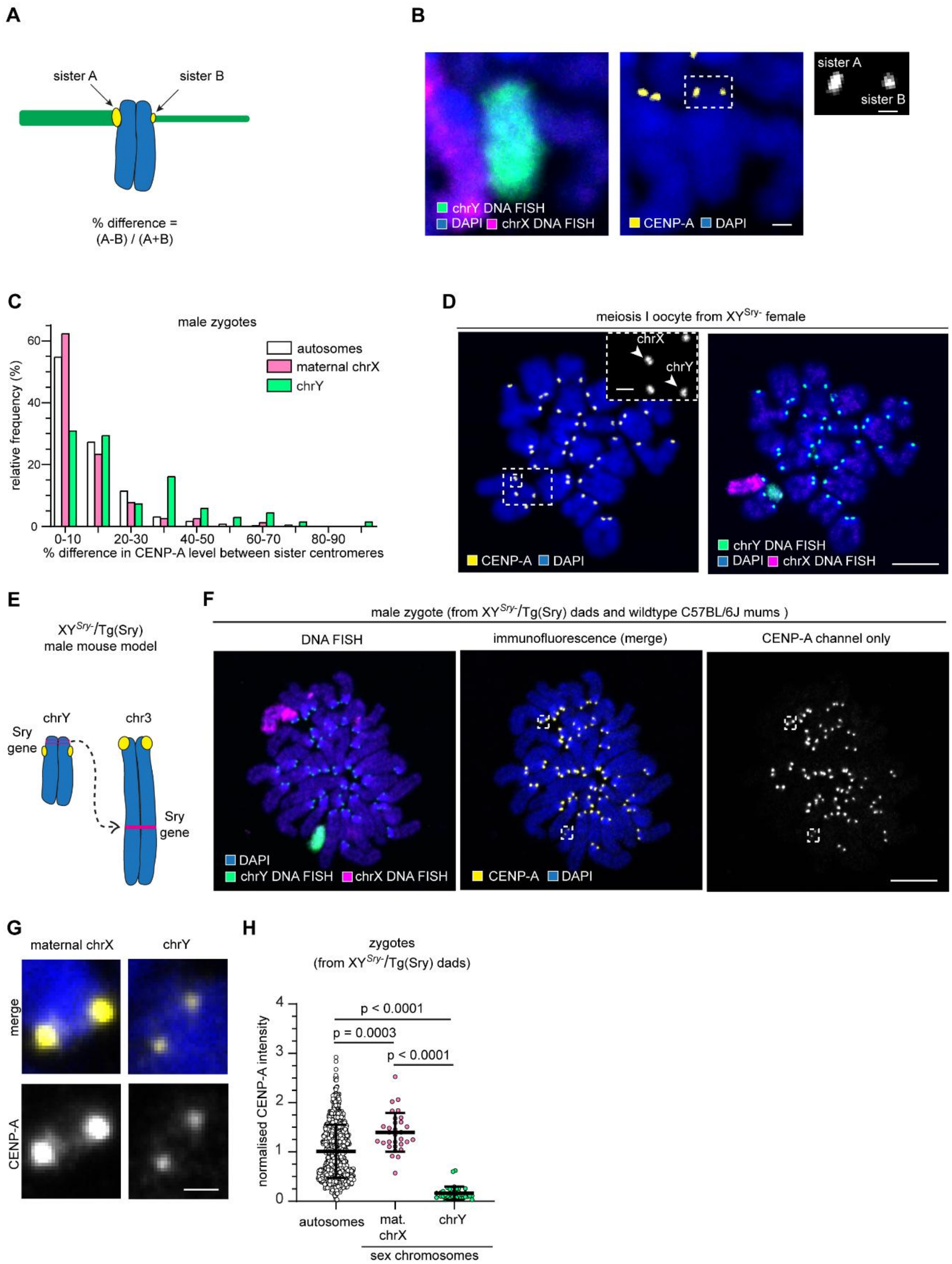

Fig. S3: Cellular context shapes the Y centromere and CENP-A is often unevenly distributed across sister chromatids. (**A**) Schematic showing how an uneven distribution of CENP-A across sister centromeres can impact kinetochore function. Formula highlights how the % difference in CENP-A level was calculated. (**B**) Maximum intensity projection of the mouse Y chromosome in

zygote, showing the sister centromeres. Left panel shows sex chromosomes labelled during DNA FISH, next panel highlights CENP-A labelling in yellow. Scalebars: large images 1 µm, insets 0.5 µm. (**C**) Frequency distribution of the differences in total CENP-A intensity between sister centromeres of chromosomes Y, maternal X, and autosomes in the zygote. Centromeres analyzed: n = 1,978 autosomes; 77 chrX; 68 chrY. (**D**) Representative maximum intensity projection of a meiosis I oocyte from a C57BL/6J strain XY^{Sry-} female. Inset shows a magnification of sex chromosome centromeres. Scalebars: 10 µm overview, 2 µm insets. (**E**) Schematic illustrating how the XY Sry^{-}/Tg(Sry) mouse model permits analysis of the Y chromosome in a male cellular context despite the absence of Sry on the Y chromosome. (**F**) Maximum intensity projection of chromosome spreads from the first embryonic metaphase of a male zygote from a XY Sry^{-}/Tg(Sry) dad. Panel on the left shows DNA FISH for sex chromosomes, while panel on the right is an image of the same zygote with CENP-A antibody labelling. Scale bar: 10 µm. (**G**) Insets show a magnification of sex chromosome centromeres from panel (F). Scale bar: 1 µm for insets. (**H**) Quantification of normalised CENP-A sum fluorescence intensity of individual centromeres in male zygotes from XY Sry^{-}/Tg(Sry) fathers. Centromeres analysed: N = 3 experimental repetitions, n = 1,342 autosomes; 32 chrX centromeres; 36 chrY centromeres. Statistical significance was evaluated using Kruskal-Wallis test. Error bars show mean +/-SD.

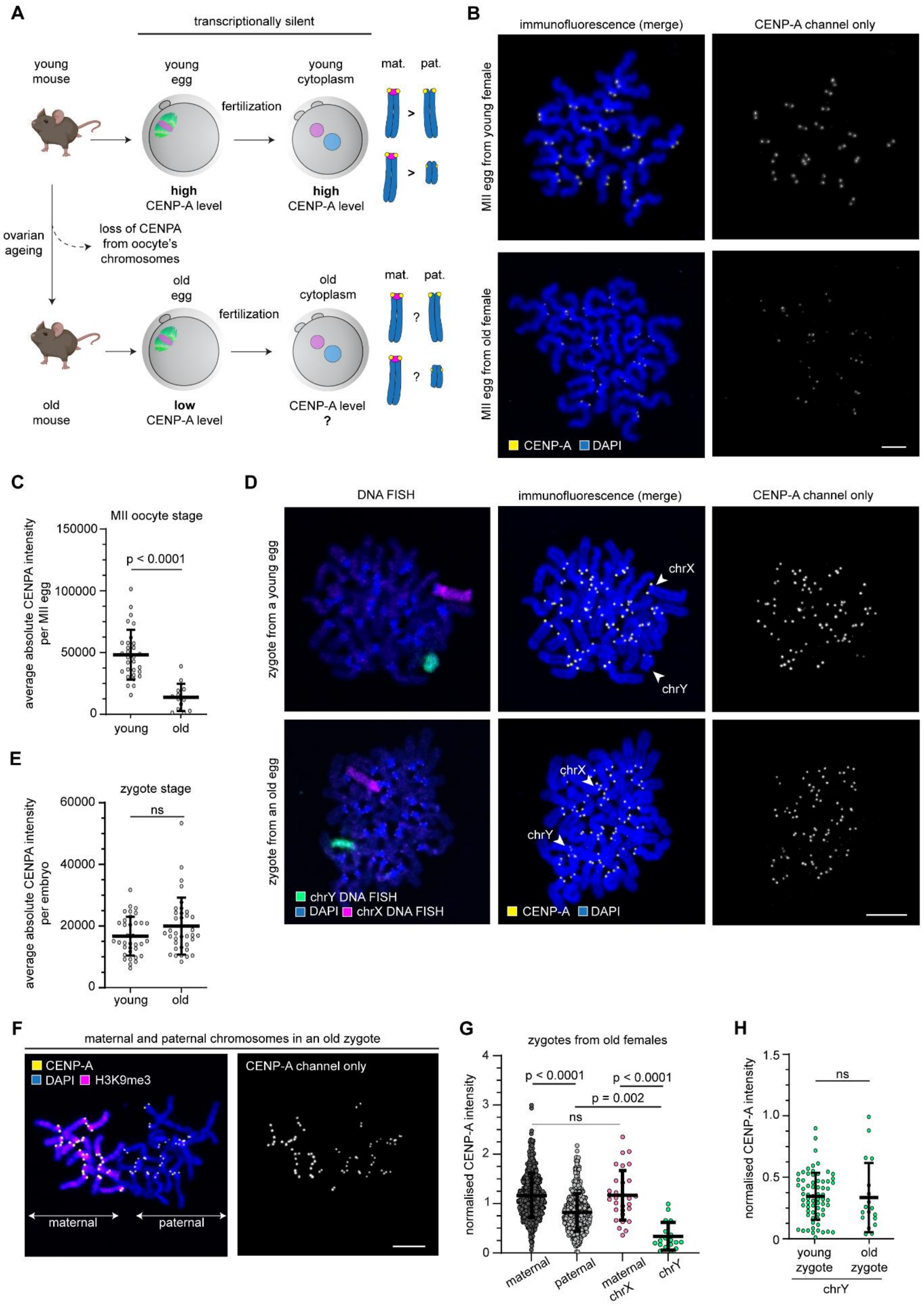

Fig. S4: The Y centromere strength in the zygote is resistant to maternal ageing. (**A**) Scheme illustrating how the maternal age-related CENP-A depletion may impact the first mitotic division of the zygote. Created in BioRender. Zielinska, A. (2026) https://BioRender.com/pt8zc63

(**B**) Maximum intensity projection of a chromosome spread of an MII egg from a young mouse (top) and an old mouse (bottom). The CENP-A signal is visualised using the same brightness and contrast parameters. (**C**) Quantification of the average absolute centromeric CENP-A intensity in MII oocytes from young versus old F1 hybrid females (C57BL/6J x CBA), acquired as shown in (B). Each dot represents the average absolute intensity across centromeres of a single MII egg. N = 3 young mice and 3 old mice processed individually, n = 30 old eggs and 13 young eggs. (**D**) Maximum intensity projection of chromosome spreads from a zygote of a young mouse (top) and an old mouse (bottom). CENP-A signal displayed using consistent brightness and contrast parameters. Arrowheads indicate the sex chromosomes, confirmed by DNA FISH. (**E**) Quantification of absolute CENP-A intensity at zygotic centromeres from young versus old mice, processed as (D). Each dot represents the average absolute intensity across centromeres of an individual zygote. N = 6 young mice and 6 old mice, processed individually. n = 35 young zygotes and 36 older zygotes. (**F**) Maximum intensity projection of a chromosome spread from a zygote of an old mouse, illustrating a notable difference in CENP-A intensity between maternal and paternal centromeres. (**G**) Quantification of normalized CENP-A sum fluorescence intensity for individual centromeres in old female zygotes from CBAxB6 F1 hybrid mothers, categorized by parental origin of the centromeres. Each dot represents a single centromere. Zygotes from 5 aged mice were processed individually: n = 703 maternal autosomes, 690 paternal autosomes, 48 sex chromosomes. (**H**) Comparison of the Y centromere's strength in young (replotted from Fig. 1G) versus old zygotes (replotted from fig. S4G). Each dot represents a single centromere. Scalebars: 10 µm. Statistical significance was evaluated using Mann-Whitney test (C, E, H) and Kruskal-Wallis test (G). Error bars show mean +/-SD.

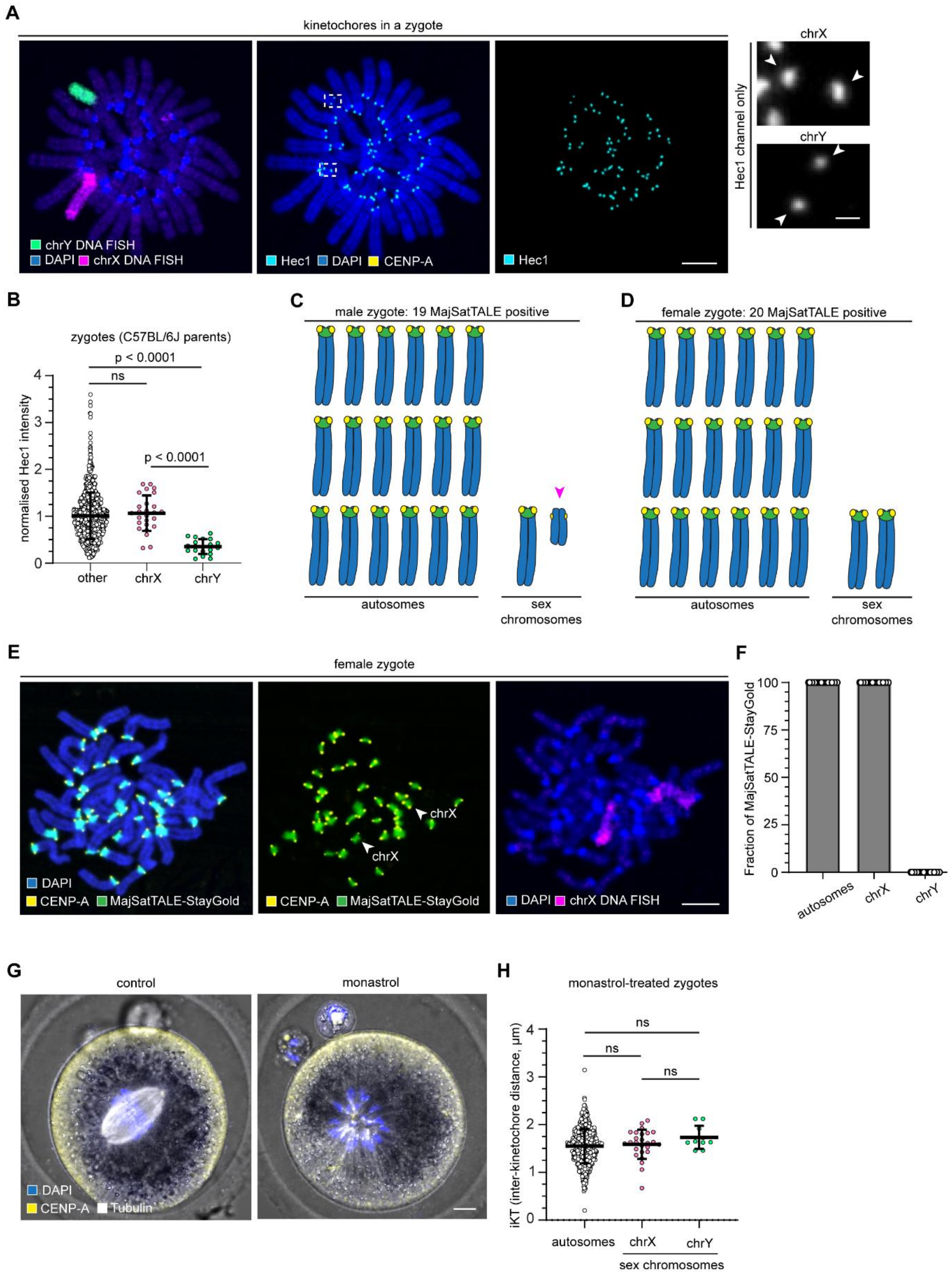

Fig. S5: The zygotic Y kinetochore weakness is conserved across strains and MajSat labelling can be used to study the Y centromere through indirect labelling.

(**A**) Representative maximum intensity projection of a male zygote from the inbred C57BL/6J strain labelled for the kinetochore protein Hec1 (cyan). (**B**) Quantification of normalised Hec1 fluorescence intensity of individual centromeres from mice processed as in (A). Each dot represents a single centromere of a male zygote. Kinetochores analysed: n = 894 autosomes; 23 chrX; 16 chrY. Statistical significance was evaluated using Kruskal-Wallis test. (**C, D**) Scheme summarising the outcome of the labelling approach shown in Fig. 3D and in fig. S5E. In male zygotes (C), 19 chromosomes are positive for MajSatTALE, while the unlabelled chromosome is the Y. In female zygotes (D), all 20 chromosomes are labelled. (**E**) Maximum intensity projection of a female C57BL/6J zygote microinjected with mRNA encoding MajSatTALE-StayGold protein (green), followed by immunofluorescence labelling for CENP-A (yellow) and DNA FISH. (**F**) Quantification of the fraction of autosomes, chrX and chrY in C57BL/6J zygotes that were MajSat positive. Samples were labelled as in Fig. 3E and in fig. S5E. N = 45 microinjected zygotes from 9 experimental repetitions, n = 1,710 autosomes, 66 X chromosomes and 24 Y chromosomes. (**G**) Overview of control (left) and monastrol-treated mouse zygotes (right), with spindles labelled in grey. (**H**) Quantification of inter-kinetochore distance when bipolar microtubule pulling is prevented, in monastrol-treated cells with unipolar spindles. Kinetochores analysed: n = 894 autosomes; 23 chrX; 16 chrY. Statistical significance was evaluated using Kruskal-Wallis test. Boxes mark regions that are magnified in insets. Arrowheads highlight individual centromeres of sex chromosomes. Scalebars: overviews 10 µm, insets 1 µm. Error bars show mean +/-SD.

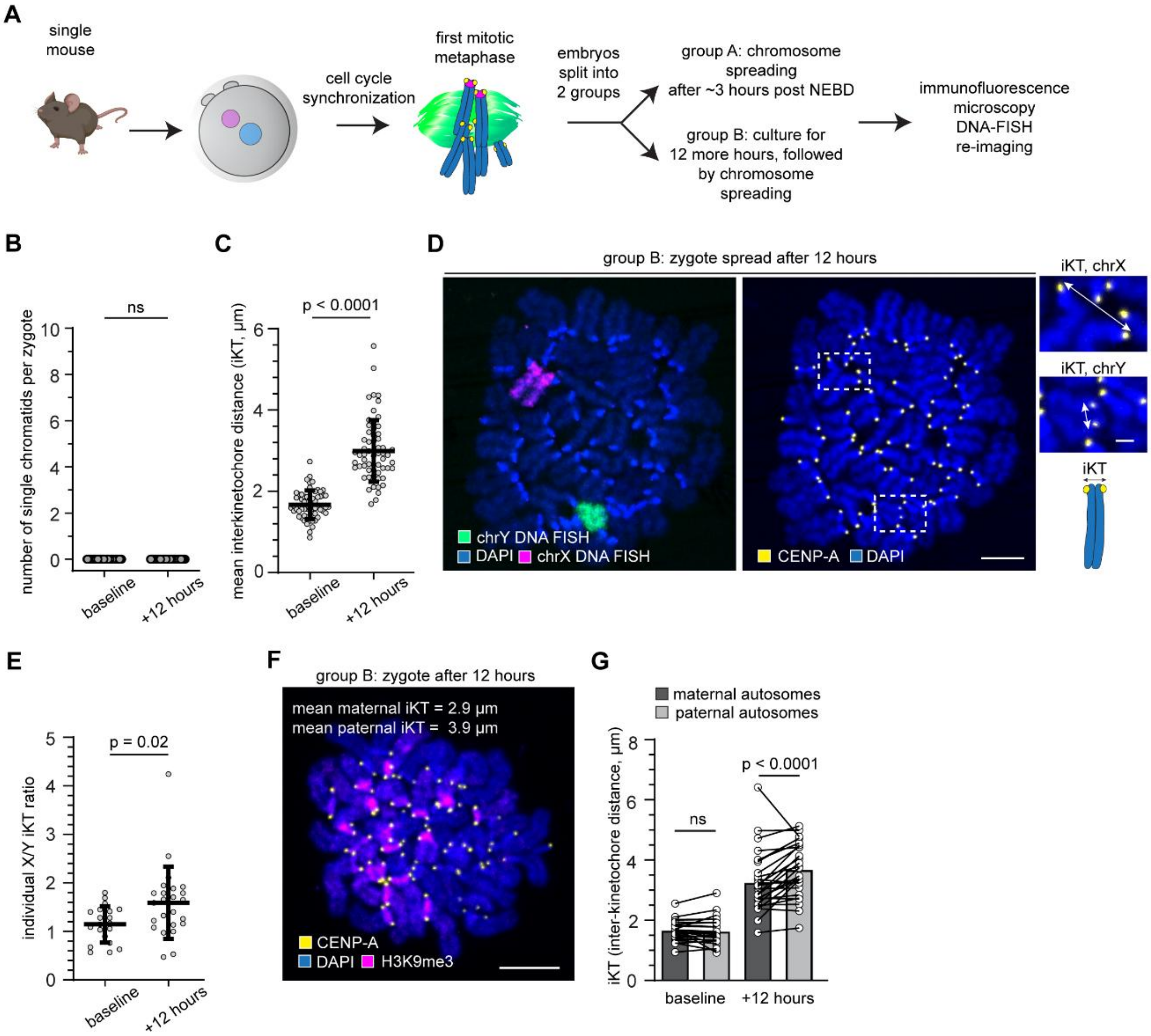

Fig. S6: The zygotic Y chromosome is less susceptible to damage from prolonged microtubule pulling, which is in contrast to an average paternal chromosome.

(**A**) Scheme representing the experimental approach to study the impact of microtubule pulling of different durations on various zygotic chromosomes from the same mouse. Created in BioRender. Zielinska, A. (2026) https://BioRender.com/pt8zc63 (**B**) Quantification of the number of single chromatids detected in zygotes processed as in (A) that were exposed to a short period of microtubule pulling (fixed in the evening of nucleus breakdown, "baseline") or microtubule pulling over a period of additional ~12 hours. Each dot represents a single zygote. (**C**) Quantification of the mean inter-kinetochore distance (iKT) in zygotes spread either at baseline or after additional 12 hours of microtubule pulling. Each dot represents a single zygote. (**D**) Maximum intensity projection of a male zygote exposed to over 12 hours of microtubule pulling. The range highlighted shows the iKT for the chrX and chrY from the same zygote. (**E**) Quantification of the chrX to chrY iKT ratio. n = 20 zygotes at baseline and 24 zygotes exposed to prolonged microtubule pulling. (**F**) Representative maximum intensity projection of a male zygote exposed to over 12 hours of microtubule pulling, where maternal and paternal chromosomes can be distinguished by H3K9me3 labelling (magenta). (**G**) Paired comparisons of maternal and paternal autosomal iKT at baseline (left) and after prolonged microtubule pulling over 12 hours (right). (B, C): n = 58 zygotes at baseline and 62 zygotes exposed to additional 12 hours of microtubule pulling. Boxes mark regions that are magnified in insets. Arrowheads highlight individual centromeres of sex chromosomes. Scalebars: overviews 10 µm,

insets 1 µm. Statistical significance was evaluated using Mann-Whitney test (C), unpaired t-test (E) and Wilcoxon test (G). Error bars show mean +/- SD.

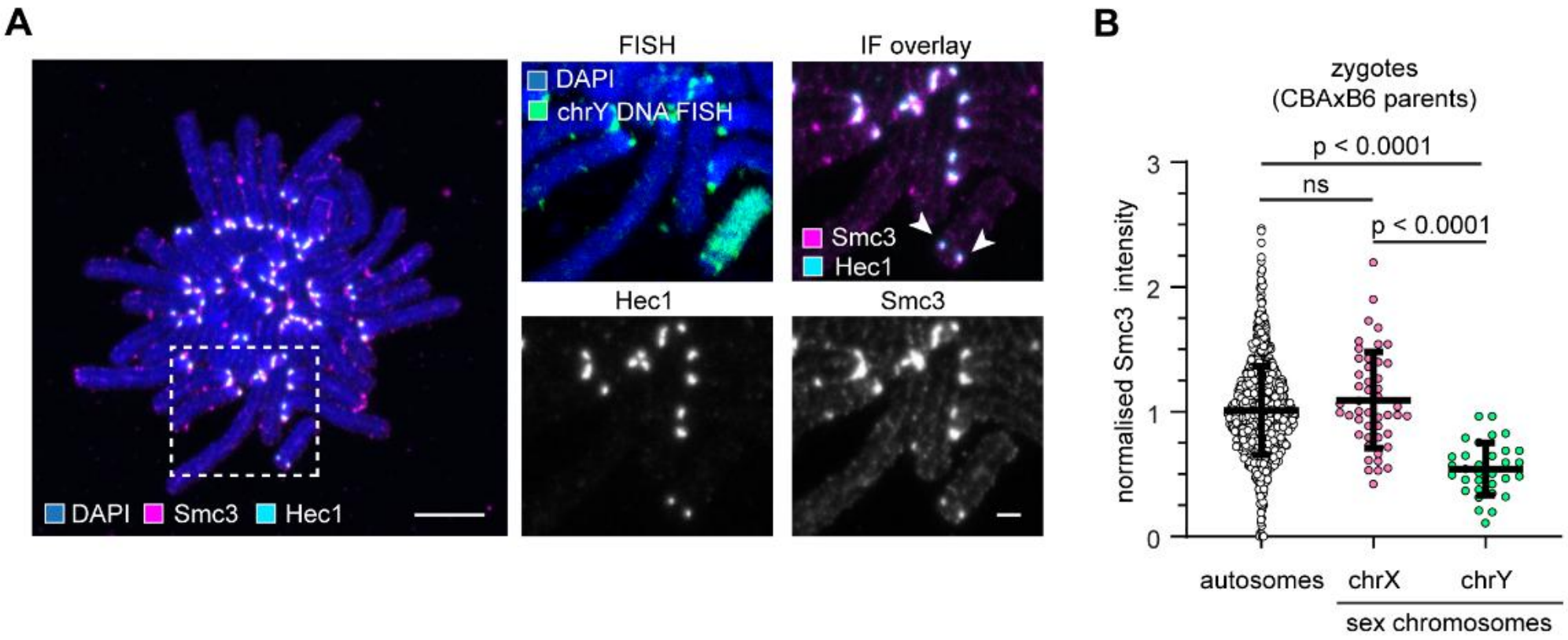

Fig. S7: The Y chromosome's resistance to cohesion fatigue is not a result of a higher absolute cohesin load at the Y centromere. (**A**) Maximum intensity projection of a male zygote from CBAxB6 F1 parents stained for cohesin subunit Smc3. The same zygote was then subjected to DNA FISH (see inset). Boxes mark regions that are magnified in insets. Arrowheads highlight the two centromeres of the Y chromosome. Scalebars: overviews 10 µm, insets 2 µm. (**B**) Quantification of normalised Smc3 sum fluorescence intensity across individual centromeres in zygotes as shown in (A). Each dot represents a single centromere. Centromeres analysed: n = 1,110 autosomes; 47 chrX; 32 chrY. Statistical significance was evaluated using Kruskal-Wallis test. Error bars show mean +/- SD.

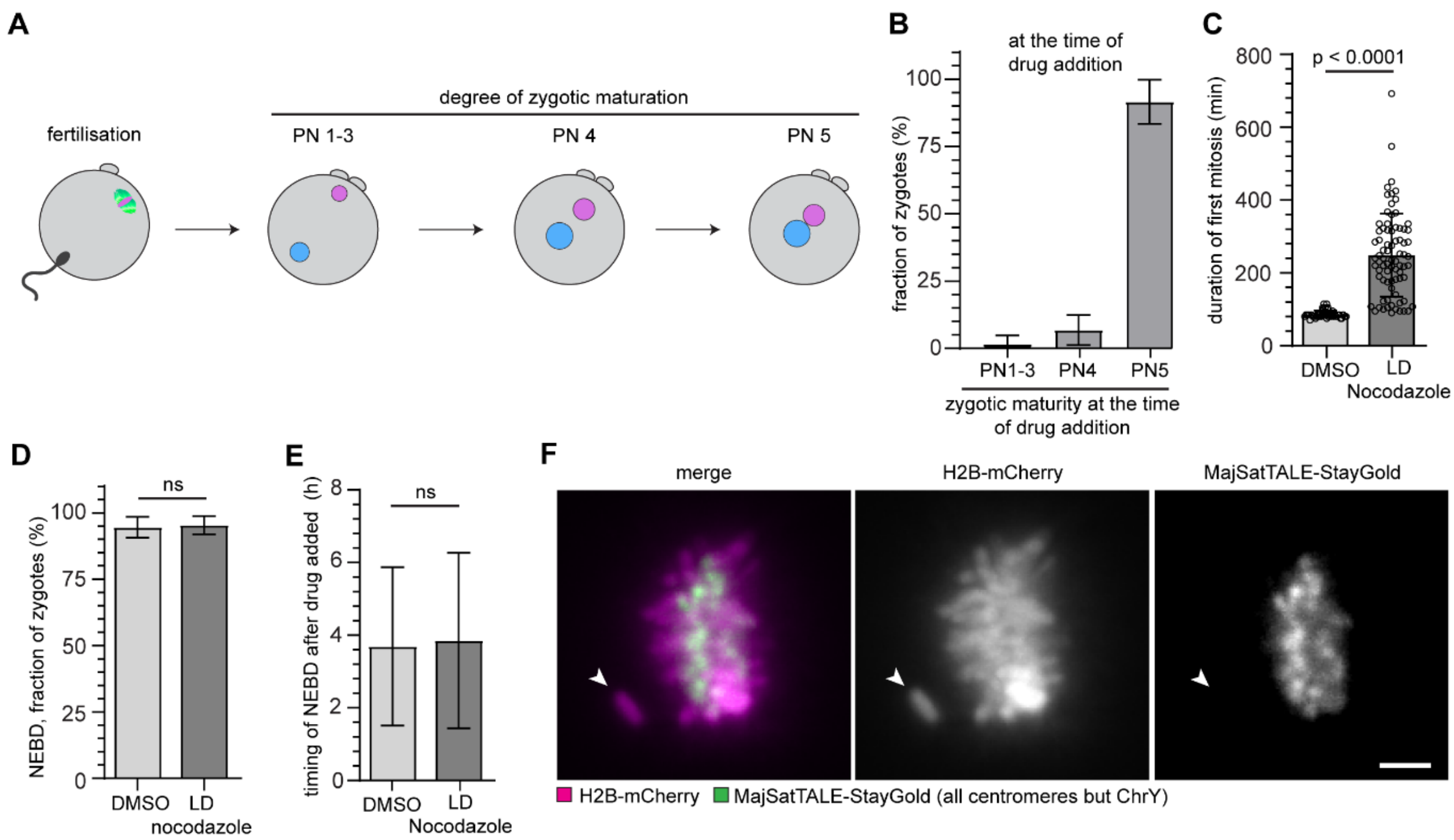

Fig. S8: Low-dose nocodazole prolongs mitosis in zygotes while allowing all cells to progress through anaphase. (**A**) Overview of pronuclear staging in the first cell cycle after fertilization. (**B**) Fraction of zygotes which were at a given pronuclear stage as described in (A) at the time of drug (DMSO/low dose (LD) nocodazole) treatment. N = 112 microinjected zygotes from 4 experimental repetitions. (**C**) Duration of the first mitosis as evaluated by live imaging (see Fig. 4A) in the control group or after addition of low dose nocodazole. Each dot represents a single embryo. N = 4 experimental repetitions, n = 48 control and 78 experimental zygotes. (**D**) Fraction of zygotes which successfully underwent nuclear envelope breakdown (NEBD) in the control group or with low dose nocodazole. N = 4 experimental repetitions, n = 63 microinjected control and 87 experimental zygotes. (**E**) Average timing of NEBD per experiment, either in the control setting or in the presence of low dose nocodazole. (**F**) Example of chrY (MajSat negative) at the spindle pole one timeframe before anaphase onset. Magenta, chromosomes (H2B-mCherry); green, centromeric repetitive sequences (MajSatTALE-StayGold). Scalebar shows 5 µm. White arrowheads point towards chrY. Statistical significance was evaluated using Mann-Whitney test (C, E) and Wilcoxon test (D). Error bars show mean +/- SD.

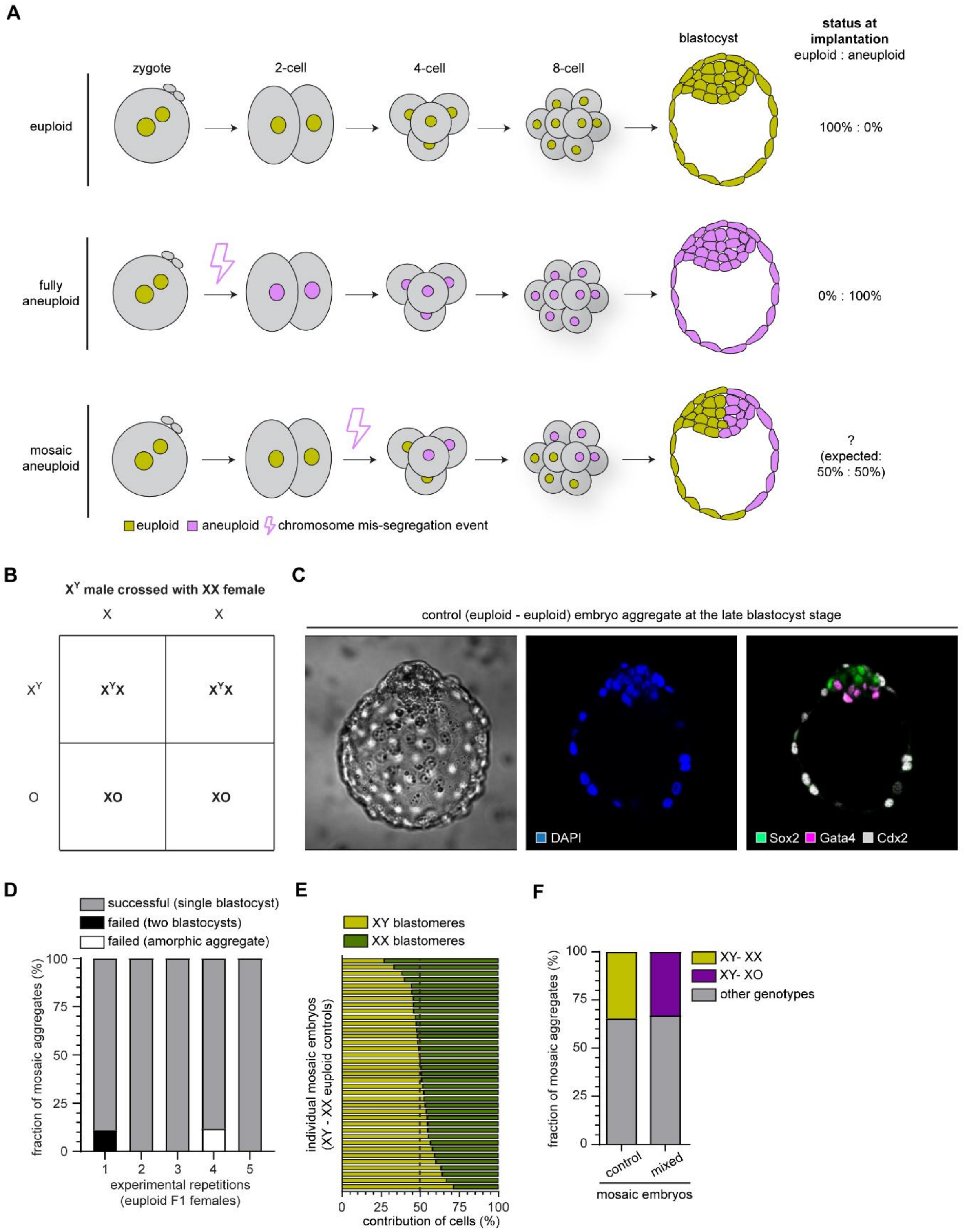

Fig. S9: Aneuploidy arising in zygotes leads to fully aneuploid blastocysts, while aneuploidy arising later in embryogenesis has the potential to be partially mitigated

(**A**) Scheme showing normal development (top row) and the outcomes for the preimplantation embryo (blastocyst) depending on the timing of the chromosome mis-segregation event (middle and bottom rows). While chromosome mis-segregation in the zygote will result in all cells of the embryo being aneuploid (middle row, magenta), if the mis-segregation error occurs in one blastomere at the 2-cell stage (bottom row), only half of the cells will be aneuploid at the 4-cell stage. This will then result in

the blastocyst being mosaic. Whether the euploid:aneuploid contribution of blastomeres continues to be 50% : 50% as the embryo grows is unknown in the context of sex chromosome aneuploidies. (**B**) Punnet square showing embryonic genotypes when an X^y male is crossed with a wild-type XX female. (**C**) Immunofluorescence image of a control mosaic blastocyst generated as described in Fig. 5A. (**D**) Efficiency of the aggregation protocol in control (XX + XX, XX + XY, XY + XY) mosaics. N = 106 aggregates from 5 experimental repetitions. (**E**) Frequency of XX and XY cells in the late blastocyst in euploid control aggregates that started as a 50% XY : 50% XX mosaics, as in (A). n = 37 XY : XX control aggregates (CBAxB6 F1 hybrid or MF1 parents). (**F**) Fraction of mosaic embryos of the desired genotype in the control group (green, XY - XX) and experimental group (purple, XY - XO). These embryos were then analysed for Fig. 5F.

Movie legends

Movie S1. Navigation through confocal sections of the same female zygote labelled with DNA FISH probes (left; X chromosomes – magenta) and antibodies for immunofluorescence (right; yellow – CENP-A, blue – DAPI, white – H3K9me3). Pink arrows point to the centromeres of the maternal X chromosome, while blue arrows point to the centromeres of the paternal X chromosome.

Movie S2. Navigation through confocal sections of the same male zygote labelled with DNA FISH probes (left; X chromosome – magenta, Y chromosome - green) and antibodies for immunofluorescence (right; yellow – CENP-A, blue – DAPI, white – H3K9me3). White arrows point to the centromeres of the sex chromosomes.

Movie S3. Navigation through a 3-D reconstruction of the first mitotic spindle in a male zygote. The zygote was injected with mRNA encoding a fluorescently labelled TALE that recognises the Major Satellite (green), and then fixed and stained with antibodies for CENP-A (yellow), tubulin (white) and DAPI. Arrows point to the only centromere that lacks the green signal (Y chromosome).

Movie S4. Time lapse movies of two zygotes progressing through the first mitosis in either control conditions (DMSO, left) or low dose nocodazole (right). The zygotes were injected with mRNA labelling histone H2B (magenta) and Major Satellite (green). Green arrows point towards chromosomes that misalign transiently.

Movie S5. Time lapse movies of two zygotes progressing through the first mitosis in either control conditions (DMSO, left) or low dose nocodazole (right). The zygotes were injected with mRNA labelling histone H2B (magenta) and Major Satellite (green). The white arrow point towards a chromosome that lacks fluorescent signal at the major satellite (Y chromosome) and permanently misaligns until anaphase onset.

Movie S6. 3-D reconstructions of a control mosaic (left, XY-XY) and a mixed mosaic (right, XY-XO). The two mosaics come from the same experimental repetition and were cultured in parallel. Images are shown using the same scaling (scalebar – 30μm).
